# Tirant Stealthily Invaded Natural *Drosophila melanogaster* Populations during the Last Century

**DOI:** 10.1093/molbev/msaa308

**Published:** 2020-11-28

**Authors:** Florian Schwarz, Filip Wierzbicki, Kirsten-André Senti, Robert Kofler

**Affiliations:** Institut für Populationsgenetik, Vetmeduni Vienna, Vienna, Austria; Vienna Graduate School of Population Genetics, Vetmeduni Vienna, Vienna, Austria; Institut für Populationsgenetik, Vetmeduni Vienna, Vienna, Austria; Vienna Graduate School of Population Genetics, Vetmeduni Vienna, Vienna, Austria; Institut für Populationsgenetik, Vetmeduni Vienna, Vienna, Austria; Institut für Populationsgenetik, Vetmeduni Vienna, Vienna, Austria

**Keywords:** transposable elements, *Drosophila melanogaster*, transposon invasions, next-generation sequencing, Tirant, P-element, I-element, hobo

## Abstract

It was long thought that solely three different transposable elements (TEs)—the I-element, the P-element, and hobo—invaded natural *Drosophila melanogaster* populations within the last century. By sequencing the “living fossils” of *Drosophila* research, that is, *D. melanogaster* strains sampled from natural populations at different time points, we show that a fourth TE, Tirant, invaded *D. melanogaster* populations during the past century. Tirant likely spread in *D. melanogaster* populations around 1938, followed by the I-element, hobo, and, lastly, the P-element. In addition to the recent insertions of the canonical Tirant, *D. melanogaster* strains harbor degraded Tirant sequences in the heterochromatin which are likely due to an ancient invasion, likely predating the split of *D. melanogaster* and *D. simulans*. These degraded insertions produce distinct piRNAs that were unable to prevent the novel Tirant invasion. In contrast to the I-element, P-element, and hobo, we did not find that Tirant induces any hybrid dysgenesis symptoms. This absence of apparent phenotypic effects may explain the late discovery of the Tirant invasion. Recent Tirant insertions were found in all investigated natural populations. Populations from Tasmania carry distinct Tirant sequences, likely due to a founder effect. By investigating the TE composition of natural populations and strains sampled at different time points, insertion site polymorphisms, piRNAs, and phenotypic effects, we provide a comprehensive study of a natural TE invasion.

## Introduction

Transposable elements (TEs) are DNA sequences that multiply within host genomes, even if this activity is deleterious to hosts (Doolittle and Sapienza 1980; Orgel and Crick 1980; Hickey 1982; Wicker et al. 2007). To enhance their rate of transmission into the next generation, TEs need to infect the germ cells. Although most TEs achieve this by being active in the germline, some LTR retrotransposons generate virus-like particles in the somatic follicle cells surrounding the germline, which may infect the germ cells (Song et al. 1997; Blumenstiel 2011; Goodier 2016; Moon et al. 2018; Wang et al. 2018). Since many TE insertions are deleterious, host organisms evolved elaborate defense mechanisms against TEs (Brennecke et al. 2007; Marí-Ordóñez et al. 2013; Yang et al. 2017). In *Drosophila melanogaster*, the defense against TEs is based on piRNAs (PIWI-interacting RNAs), that is, small RNAs with a size between 23–29nt, that repress TE activity at the transcriptional and the posttranscriptional level (Brennecke et al. 2007; Gunawardane et al. 2007; Sienski et al. 2012; Le Thomas et al. 2013). piRNAs are derived from distinct genomic loci termed piRNA clusters (Brennecke et al. 2007). Different piRNA pathways are active in the germline and in the follicle cells surrounding the germline (Li, Vagin, et al. 2009; Malone et al. 2009), where solely the germline pathway depends on maternally transmitted piRNAs for efficient silencing of TEs (Le Thomas et al. 2014).

One option to escape the host defense is to infect a novel species. Many TEs cross species boundaries, for example, due to horizontal transfer (HT) from one host species to another, and trigger invasions in naive species not having the TE (Mizrokhi and Mazo 1990; Maruyama and Hartl 1991; Lohe et al. 1995; Terzian et al. 2000; Sánchez-Gracia et al. 2005; Loreto et al. 2008; Kofler, Hill, et al. 2015; Peccoud et al. 2017). A striking example for a high frequency of TE invasions can be seen in *D. melanogaster*, which was invaded by at least three different TE families within the last century: the I-element, hobo, and the P-element (Kidwell 1983; Anxolabéhère et al. 1988; Periquet et al. 1989; Daniels, Chovnick, et al. 1990; Daniels, Peterson, et al. 1990; Bucheton et al. 1992; Bonnivard et al. 2000). All of these three TEs actively replicate only in the germline and induce some phenotypic effects, the hybrid dysgenesis (HD) symptoms, which historically led to the discovery of the recent TE invasions in *D. melanogaster* (Bingham et al. 1982; Calvi and Gelbart 1994; Biémont 2010; Moon et al. 2018; Wang et al. 2018). An important hallmark of these HD symptoms is that the direction of crosses between two strains is important. The offspring of crosses between males carrying a genomic factor (the TE) and females not carrying this factor frequently show various symptoms (e.g., atrophic ovaries) whereas the offspring of the reciprocal crosses is usually free of symptoms (Bucheton et al. 1976; Kidwell et al. 1977; Blackman et al. 1987; Yannopoulos et al. 1987). Hence, hybrid dysgenesis has a cytoplasmic as well as a genomic component.

Although TEs were quickly identified as the responsible genomic factor, the cytoplasmic component, the maternally transmitted piRNAs, was discovered much later (Bingham et al. 1982; Brennecke et al. 2008). It was realized that the presence of an HD-inducing TE in a strain mostly depends on the sampling date of a strain, where more recently sampled strains frequently carry the TE while old strains, sampled before the invasion, do not. It was thus suggested that the HD-inducing TEs recently invaded *D. melanogaster* populations (Kidwell 1983; Periquet et al. 1994). These invasions were probably triggered by HT events, where the P-element was likely acquired from *D. willistoni* and the I-element as well as hobo possibly from *D. simulans* (or another species from the *simulans* clade) (Daniels, Chovnick, et al. 1990; Daniels, Peterson, et al. 1990; Simmons 1992; Loreto et al. 2008; Blumenstiel 2019). However, even the old strains carried short and highly degraded (probably inactive) fragments of the I-element and hobo, mostly in the heterochromatin (Bucheton et al. 1984, 1986, 1992; Daniels, Chovnick, et al. 1990). Hence, the I-element and hobo likely invaded *D. melanogaster* populations at least twice. Solely the P-element does not have substantial similarity to sequences in the *D. melanogaster* genome, which suggests that the P-element invaded *D. melanogaster* populations for the first time. *Drosophila melanogaster* strains sampled at different time points, previously labeled as the “living fossils” of *Drosophila* research (Bucheton et al. 1992), were not only used to discover the three recent TE invasions but also to estimate the timing of the invasions: the I-element invasion occurred presumably between 1930 and 1950, the hobo invasion around 1955 and the P-element invasion between 1950 and 1980 (Kidwell 1983; Anxolabéhère et al. 1988; Periquet et al. 1989).

By sequencing these “living fossils,” we discovered that an additional transposon, Tirant, invaded *D. melanogaster* populations within the last century. Previous work showed that Tirant is an LTR retrotransposon and a member of the *Ty3/Gypsy* superfamily (Moltó et al. 1996; Viggiano et al. 1997; Cañizares et al. 2000; Terzian et al. 2001). It encodes an envelope protein and completes the retroviral cycle in the closely related *D. simulans* (Lemeunier et al. 1976; Marsano et al. 2000; Akkouche et al. 2012). In contrast to the P-element, hobo, and the I-element, which are active in the germline, Tirant was classified as an intermediate TE based on the amount of maternally transmitted piRNAs, that is, Tirant is likely expressed and targeted in both the germline and in somatic follicle cells (Malone et al. 2009). In agreement with this, Tirant activity was reported in both tissues (Akkouche et al. 2012). Furthermore, knockdowns of components of the germline as well as the somatic piRNA pathway, result in a reduction of Tirant piRNAs (Nefedova et al. 2012; Czech et al. 2013; Rozhkov et al. 2013; Barckmann et al. 2018). Generally, intermediate TEs are little understood. However, for Tirant in particular, peculiarities in the regulation were noted (Akkouche et al. 2013; Parhad et al. 2017; Wang et al. 2020). For example, in some backgrounds Tirant may be upregulated independent of piRNAs (Parhad et al. 2017).

Fablet et al. (2007) suggested that Tirant is an ancient TE that is largely vertically transmitted in the *D. melanogaster* species subgroup. Analyses of the reference genome of *D. melanogaster* revealed the presence of degraded Tirant insertions in the heterochromatin and full-length insertions in the euchromatin (Bowen and McDonald 2001; Mugnier et al. 2008). The heterochromatic insertions are likely ancient, possibly predating the split of *D. melanogaster* and *D. simulans*, whereas the euchromatic insertions are likely more recent (<16,000–200,000 years) (Bowen and McDonald 2001; Bergman and Bensasson 2007; Mugnier et al. 2008). This raises the question on how this uneven age distribution of Tirant insertions evolved.

Here, we show that full-length (canonical) Tirant sequences are absent from laboratory strains sampled before 1938 but present in strains sampled after 1938. We thus suggest that the canonical Tirant invaded natural *D. melanogaster* populations between 1930 and 1950, possibly following an HT from *D. simulans*. This invasion constitutes a second wave of activity, with degraded heterochromatic fragments being the remnants of an ancient Tirant invasion, possibly in the ancestor of the *D. melanogaster* species subgroup. Tirant is thus the fourth TE to invade *D. melanogaster* populations within the last century. Based on a consistent approach (i.e., the same method and strains) for all four TEs, we estimate that Tirant invaded *D. melanogaster* populations first, followed by the I-element, hobo and, finally, the P-element. Recent Tirant insertions were found in all investigated natural populations, where populations from Tasmania carry distinct Tirant sequences, likely due to a founder effect.

Although all strains carry piRNAs complementary to the degraded Tirant insertions solely recently invaded strains carry piRNAs complementary to the canonical Tirant. We thus suggest that piRNAs complementary to heterochromatic insertions were too diverged to prevent the spread of the canonical Tirant. Finally, we did not find apparent HD symptoms induced by Tirant, which may account for the late discovery of the Tirant invasion. By investigating the TE composition (i.e., abundance of TEs and frequency of internal deletions and SNPs) of natural populations and strains sampled at different time points, insertion site polymorphisms, piRNAs, and phenotypic effects, we provide a comprehensive study of a natural TE invasion.

## Results

### Canonical Tirant Insertions Are Present in Iso-1 but Not in Canton-S

Given the striking accumulation of TE invasions within the last century (Kidwell 1983; Anxolabéhère et al. 1988; Periquet et al. 1989; Daniels, Chovnick, et al. 1990; Daniels, Peterson, et al. 1990; Bucheton et al. 1992; Bonnivard et al. 2000), we speculated that additional, hitherto undetected TEs, may have recently invaded *D. melanogaster* populations.

**Fig. 1.**
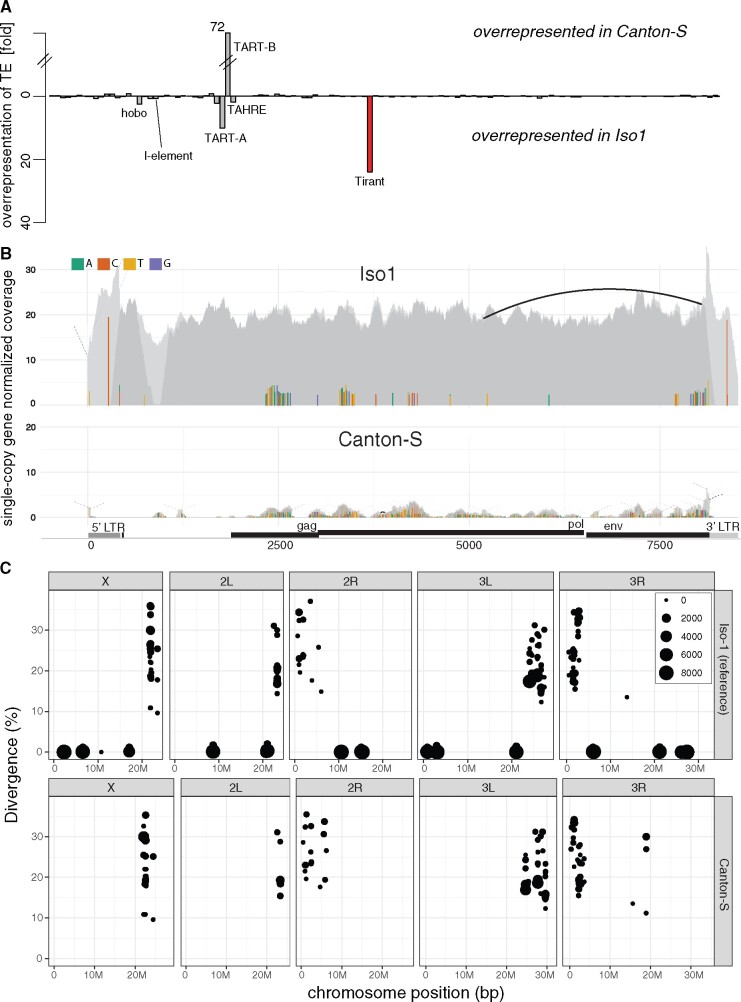
Canonical Tirant insertions are present in Iso-1 but not Canton-S. (*A*) Differences in TE content between Canton-S and Iso-1. For each TE family (*x* axis), we show the fold-difference in the number of reads mapping to a TE (*y* axis) between the two strains. Note that reads mapping to Tirant (red) are overrepresented in Iso-1. (*B*) Abundance and diversity of Tirant in Iso-1 and Canton-S. Short reads were aligned to the consensus sequence of Tirant and visualized with DeviaTE. The coverage of Tirant was normalized to the coverage of single-copy genes. Single-nucleotide polymorphisms (SNPs) and small internal deletions (indels) are shown as colored lines. Large internal deletions are shown as black arcs (the frequency of the shown deletion is ≈5%). Coverage based on uniquely and ambiguously mapped reads is shown in dark and light gray, respectively. Note that solely a few, highly degraded copies of Tirant are present in Canton-S. (*C*) Overview of Tirant insertions in the genomes of Iso-1 and Canton-S. For each Tirant insertion, we show the position in the assembly, the length (size of dot), and the similarity to the consensus sequence (divergence).

To test this hypothesis, we compared the abundance of TEs between one of the oldest available *D. melanogaster* laboratory strains, Canton-S (collected by C. Bridges in 1935; Lindsley and Grell 1968) and the reference strain, Iso-1 (fig. 1*A*; Brizuela et al. 1994). We aligned publicly available short-read data from these strains to the consensus sequences of TEs in *D. melanogaster* (Quesneville et al. 2005) and estimated the normalized abundance (reads per million) of the TEs in these two strains with our novel tool DeviaTE (Weilguny and Kofler 2019). Apart from the telomeric TEs (TART-A, TART-B, and TAHRE) which show distinct evolutionary dynamics (Pardue and DeBaryshe 2011; Saint-Leandre and Levine 2020), the most striking difference between the two strains was due to the LTR retrotransposon Tirant (fig. 1*A*). As expected, hobo and the I-element, two TEs that invaded *D. melanogaster* recently, are more abundant in the Iso-1 strain than in the older Canton-S strain (fig. 1*A*). The P-element is not present in both strains. To further investigate the abundance of Tirant in the two strains, we calculated the coverage of reads along the Tirant sequence with DeviaTE (fig. 1*B*; Weilguny and Kofler 2019). We observed striking coverage differences between Canton-S and Iso-1 over the entire sequence of Tirant (fig. 1*B*; average normalized coverage; Iso-1 = 20.9, Canton-S = 0.86). Only few highly diverged reads aligned to Tirant in Canton-S (fig. 1*B*). In addition to these diverged reads, many reads with a high similarity to the consensus sequence of Tirant aligned in Iso-1 (fig. 1*B*). We refer to Tirant sequences with a high similarity to the consensus sequence as “canonical” Tirant. To identify the genomic location of the canonical and the diverged Tirant sequences, we annotated TEs in publicly available assemblies of Canton-S (based on Oxford Nanopore long-read data) and Iso-1 (i.e., the reference genome) with RepeatMasker (fig. 1*C*; Hoskins et al. 2015; Wierzbicki et al. 2020). Both assemblies are of high quality and suitable for genomic analysis of TEs (Wierzbicki et al. 2020). In Canton-S, only highly fragmented and diverged Tirant sequences were found close to the centromeres (fig. 1*C* and supplementary fig. 1, Supplementary Material online). In addition to these diverged Tirant sequences, Iso-1 carries several canonical Tirant insertions on each chromosome arm (fig. 1*C*). This genomic distribution of Tirant, that is, degraded Tirant fragments in the heterochromatin and canonical insertions in the euchromatin of *D. melanogaster*, was also noted in previous studies (Marsano et al. 2000; Mugnier et al. 2008). The absence of canonical Tirant insertions in euchromatin is also found in an independent assembly of Canton-S which is based on PacBio reads (supplementary fig. 2, Supplementary Material online; Chakraborty et al. 2019). It was proposed that the degraded Tirant insertions located in heterochromatin are ancient and likely vertically inherited from the ancestor of the *D. melanogaster* species subgroup (Moltó et al. 1996; Fablet et al. 2007; Mugnier et al. 2008). It was further proposed that canonical insertions in Iso-1 are of more recent origin (i.e., <16,000–200,000 years (Bowen and McDonald 2001; Bergman and Bensasson 2007; Lerat et al. 2011; Rahman et al. 2015). We thus speculated that the canonical insertions of Tirant may have recently been active, whereas the degraded insertions in the heterochromatic may be inactive for some time (see also, Mugnier et al. 2008; Fablet et al. 2009). If this is true, canonical insertions ought to segregate at low frequency in natural populations, whereas the degraded insertions should mostly be fixed. To test this hypothesis, we estimated the population frequencies of the canonical and the degraded Tirant insertions in a natural *D. melanogaster* population from France (Viltain) (Kapun et al. 2020) with PoPoolationTE2 (Kofler et al. 2016). Indeed, most canonical Tirant insertions segregate at a low population frequency (*f *=* *0.063) in the euchromatin, whereas most degraded insertions are in the heterochromatin and segregate at significantly higher frequencies (*f *=* *0.73; Wilcoxon rank sum test *P* < 2.2e–16; supplementary fig. 3, Supplementary Material online). Due to relaxed purifying selection in low-recombining regions (Eanes et al. 1992; Sniegowski and Charlesworth 1994; Bartolomé et al. 2002; Petrov et al. 2011; Kofler et al. 2012), degraded Tirant insertions may have accumulated in the heterochromatin. Taken together, we hypothesize that Tirant invaded natural *D. melanogaster* populations in at least two waves of activity: an ancient wave, possibly predating the split of *D. melanogaster* and *D. simulans*, and a recent wave after Canton-S was sampled.

### Canonical Tirant Invaded *D. melanogaster* Populations between 1930 and 1950

**Fig. 2.**
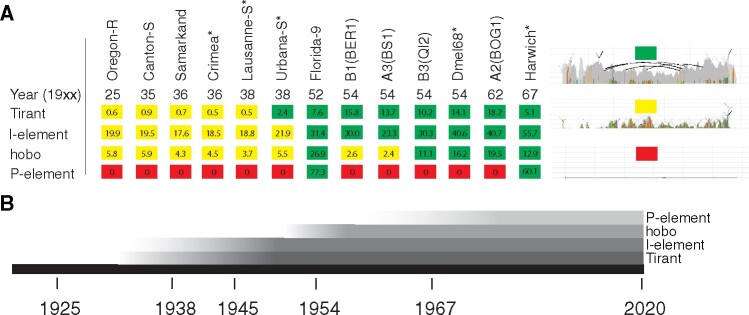
History of recent TE invasions in natural *Drosophila melanogaster* populations. (*A*) Overview of Tirant, I-element, hobo, and P-element sequences in some *D. melanogaster* strains. For an overview of these TEs in all investigated strains, see supplementary table 1, Supplementary Material online. The year corresponds to the sampling date of the strain. Strains sequenced in this work are marked by a star (*). For each family, we classified the TE content into three distinct categories: red, absence of any TE sequence; yellow, solely degraded TE sequences are present; green, nondegraded sequences, with a high similarity to the consensus sequence are present; Numbers represent estimates of TE copy numbers per haploid genome obtained with DeviaTE. The abundance of degraded copies may be underestimated as copy-number estimates are based on the average coverage of the consensus sequence. The right panel shows an example for each of the three categories (similar to fig. 1*B*). (*B*) Timeline showing the estimated invasion history of Tirant, the I-element, hobo, and the P-element.

If Tirant invaded natural *D. melanogaster* populations recently, old strains should only have a few highly degraded Tirant sequences (similar to Canton-S), whereas more recently collected strains should have many insertions with a high similarity to the consensus sequence of Tirant (i.e., canonical Tirant insertions). To test this, we sequenced 12 of the oldest available *D. melanogaster* strains (sampled between 1920 and 1970; fig. 2; supplementary table 1, Supplementary Material online). Additionally, we included publicly available data of 15 different *D. melanogaster* strains into the analyses (fig. 2*A* and supplementary table 1, Supplementary Material online). The reads were mapped to the consensus sequences of TEs in *Drosophila* and the TE abundance was assessed with DeviaTE (supplementary fig. 4, Supplementary Material online; Weilguny and Kofler 2019).

Strikingly, six out of seven strains sampled before or in 1938 solely contained degraded Tirant sequences (supplementary table 1 and fig. 4, Supplementary Material online). The first strain carrying canonical Tirant sequences (Urbana-S) was collected around 1938. All 16 strains collected around or after 1950 carried canonical Tirant sequences (supplementary table 1, Supplementary Material online). Estimates of the TE copy numbers support these observations (fig. 2*A*). To obtain estimates of the TE abundance independent of DeviaTE, we also computed the normalized number of reads mapping to each TE (rpm; reads per million). These data also support the sudden increase in reads mapping to Tirant in strains sampled after 1938 (supplementary table 2, Supplementary Material online). We note that the raw abundance of reads mapping to a TE is highly correlated with the estimates of TE abundance obtained with DeviaTE (supplementary fig. 5, Supplementary Material online). Our results thus suggest that the canonical Tirant invaded *D. melanogaster* populations between 1938 and 1950 (fig. 2). Since we were interested in the timing of the Tirant invasion relative to the other three TEs that recently invaded *D. melanogaster* populations, we also investigated the abundance and diversity of the I-element, hobo, and the P-element in these strains (supplementary table 1 and figs. 6–8, Supplementary Material online). Our data suggest that Tirant invaded natural *D. melanogaster* populations just before the I-element, followed by hobo and, lastly, by the P-element (supplementary tables 1 and 2, Supplementary Material online and fig. 2*B*).

### Canonical Tirant Insertions Are Found in Worldwide Populations of *D. melanogaster* and Populations from Tasmania Carry Distinct Tirant Variants

**Fig. 3.**
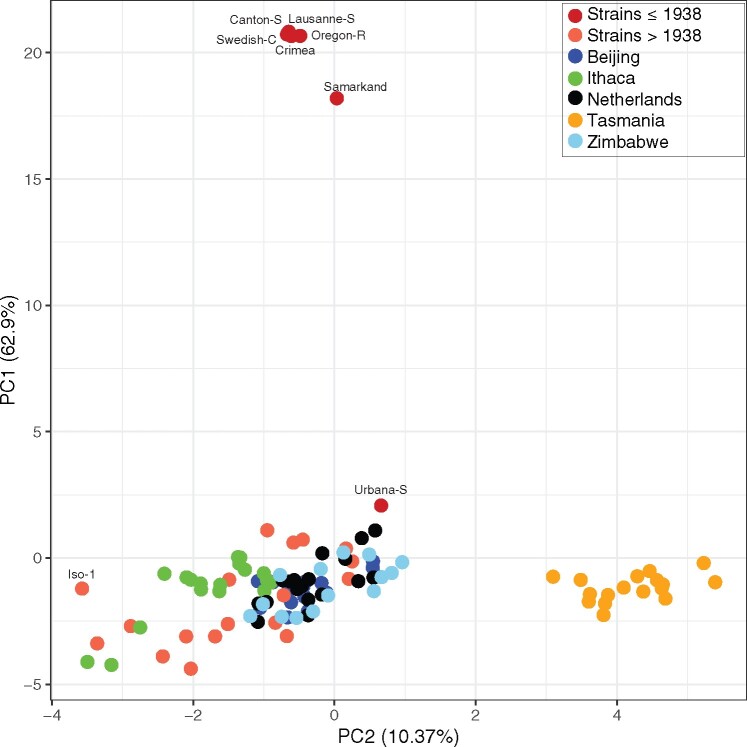
PCA based on the allele frequencies of Tirant SNPs in different *Drosophila melanogaster* strains. In addition to previously described *D. melanogaster* strains, the Global Diversity Lines (GDL) were analyzed. Note that the strains without canonical Tirant insertions as well as populations from Tasmania form distinct groups.

To further investigate the Tirant composition among strains, we performed a PCA based on the allele frequencies of Tirant single-nucleotide polymorphism (SNPs) (fig. 3). Note that our usage of the term SNP is not strictly identical to the common usage describing allelic variants at a single locus. Here, a SNP describes a variant among dispersed Tirant copies. Our allele frequency estimates thus reflect the Tirant composition within a particular strain (e.g., if 14 Tirant insertions in a given strain carry an “A” at some site and 6 a “T,” the frequency of “A” at this site is 0.7). In addition to the above-mentioned strains (supplementary table 1, Supplementary Material online), we also analyzed the Tirant content of natural populations. To do this, we relied on the global diversity lines (GDL), that is, several *D. melanogaster* strains sampled after 1988 (Begun and Aquadro 1995) from five different continents (Africa—Zimbabwe, Asia—Beijing, Australia—Tasmania, Europe—Netherlands, America—Ithaca; Grenier et al. 2015).

Old strains, collected before 1938, formed a distinct group (fig. 3), supporting our view that they carry distinct Tirant sequences. By contrast, most strains collected after 1938 and the majority of the GDLs group into one large cluster (fig. 3). All GDL strains thus carry nondegraded Tirant sequences. This observation also holds when additional, recently collected *D. melanogaster* strains are analyzed (e.g., DGRP, DrosEU, DrosRTEC; supplementary fig. 9, Supplementary Material online; Mackay et al. 2012; Bergland et al. 2014; Lack et al. 2015; Machado et al. 2019; Kapun et al. 2020). Old strains also form a distinct group in an unrooted tree computed from pairwise *F*_ST_ values based on the frequency of Tirant SNPs (supplementary fig. 10, Supplementary Material online). Our data thus suggests that Tirant invaded most worldwide *D. melanogaster* populations. The reference strain Iso-1 is distant to the large cluster (fig. 3). Closer inspection revealed that Tirant insertions from natural populations carry eight SNPs that are not found in the reference strain (supplementary fig. 11 and table 3, Supplementary Material online). Interestingly, also strains collected from Tasmania (Australia) formed a distinct group (fig. 3 and supplementary fig. 10, Supplementary Material online). We hypothesized that this is due to multiple SNPs having markedly different allele frequencies in Tasmanian populations than in populations from other geographic locations (supplementary fig. 12 and table 4, Supplementary Material online). Indeed, when excluding those SNPs from the PCA, strains from Tasmania clustered with strains sampled from the other locations (supplementary fig. 13, Supplementary Material online). For hobo and the I-element, Tasmanian populations did not form a separate cluster (supplementary fig. 14, Supplementary Material online; the P-element is absent in many samples, hence allele frequencies could not be calculated). This raises the question of what processes could be responsible for such striking differences in the Tirant composition among natural populations. We suggest that the Tirant invasion in Tasmania was subject to a founder effect, where flies carrying some rare variants of Tirant migrated to Tasmania, thereby triggering the spread of these rare Tirant variants in Tasmanian populations. Similarly, the strains used for generating Iso-1 may have carried rare Tirant variants that multiplied in these lines after they were sampled. In agreement with this, most Iso-1 specific SNPs segregate at low frequency in some *D. melanogaster* populations from Europe and North America (supplementary fig. 11, Supplementary Material online).

In summary, we conclude that Tirant invaded all investigated worldwide populations of *D. melanogaster* during the past century. Furthermore, founder effects may be important components of TE invasions, since they may lead to a geographically heterogeneous TE composition.

### The Canonical Tirant Is Silenced by a piRNA-Based Defense Mechanism

**Fig. 4.**
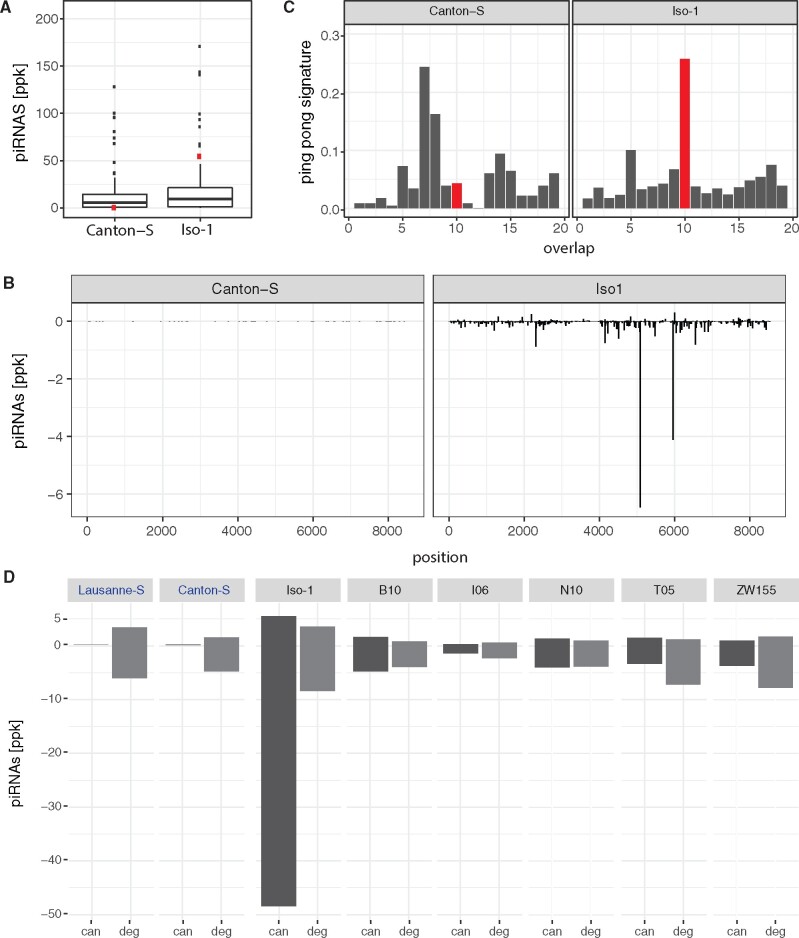
Tirant piRNAs in strains with (e.g., Iso-1) and without (e.g., Canton-S) canonical Tirant insertions. (*A*) Abundance of canonical Tirant piRNAs (red) compared with piRNA of the other TEs of *Drosophila melanogaster*. (*B*) Abundance of piRNAs along the canonical sequence of Tirant. (*C*) Ping-pong signature for canonical Tirant piRNAs. A pronounced peak at position 10 (red) suggests secondary amplification of piRNAs by the ping-pong cycle. (*D*) Abundance of piRNAs complementary to canonical (can; dark gray) and degraded (deg; light gray) Tirant sequences in laboratory strains (Lausanne-S, Canton-S, and Iso-1) and GDL lines (B10, Beijing; I06, Ithaca; N10, Netherlands; T05, Tasmania; ZW155, Zimbabwe; Grenier et al. 2015). The names of two strains not having canonical Tirant insertions are shown in blue. Sense piRNAs are on the positive *y* axis and antisense piRNAs on the negative *y* axis (*B* and *D*). ppk, piRNAs per 1000 miRNAs.

If Tirant recently invaded *D. melanogaster* populations, we expect to see differences in the composition of piRNAs between strains sampled before and after the invasion. Strains invaded by Tirant, such as Iso-1, should have established a functional defense against the TE and thus generate large amounts of piRNAs complementary to canonical Tirant. By contrast, naive strains, such as Canton-S, should have few canonical Tirant piRNAs. To test this, we sequenced piRNAs from the ovaries of both strains. Indeed, piRNAs against canonical Tirant were highly abundant in Iso-1 but not in Canton-S (fig. 4*A* and *D*). Compared with the piRNA abundance of other TE families in *D. melanogaster*, Tirant piRNAs rank among the most abundant in Iso-1 but the least abundant in Canton-S (fig. 4*A*). Both sense and antisense piRNAs are distributed over the entire sequence of Tirant in Iso-1 (fig. 4*B*). TEs that are silenced in the germline by dual-strand clusters show a characteristic 10 nt overlap between sense and antisense piRNAs, that is, the ping-pong signature (Brennecke et al. 2007; Malone et al. 2009). Tirant has a pronounced ping-pong signature in Iso-1 but not in Canton-S (fig. 4*C*), consistent with Tirant being silenced in the germline (likely in addition to the soma) (Malone et al. 2009). Finally, we wondered whether the ancient Tirant invasion, responsible for the degraded Tirant fragments in the heterochromatin, led to piRNAs against Tirant. Both Iso-1 and Canton-S, carry piRNAs complementary to the degraded Tirant fragments (6252.0 ppm in Canton-S and 11886.0 ppm in Iso-1; fig. 4*D*). An analysis of the piRNA content of additional strains (Lausanne-S and GDL lines; Luo et al. 2020) confirms that all investigated strains carry piRNAs complementary to the degraded Tirant whereas only strains with canonical Tirant insertions carry piRNAs complementary to the canonical Tirant (fig. 4*D*). This raises the question why these piRNAs of the degraded Tirant were unable to prevent the invasion of the canonical Tirant. Previous works suggest that piRNAs need to match over the bulk of a sequence with a sequence divergence of less than 10% for efficient silencing of the target sequence (Post et al. 2014; Kotov et al. 2019). Heterochromatic Tirant sequences, however, are about 10–30% diverged from the canonical Tirant (supplementary fig. 1, Supplementary Material online). The high divergence can be found over the entire sequence of these Tirant fragments (supplementary fig. 15, Supplementary Material online). Consequently very few of the degraded piRNAs match to the canonical Tirant with a sequence divergence of less than 10% (supplementary fig. 16, Supplementary Material online).

We conclude that a piRNA-based defense mechanism against the canonical Tirant is present in all strains carrying canonical Tirant insertions but absent in strains solely having heterochromatic Tirant insertions. Although piRNAs derived from these heterochromatic Tirant fragments are present in all strains, these piRNAs were likely too diverged to silence the canonical Tirant and therefore could not prevent its recent invasion.

### No Apparent Hybrid Dysgenesis Symptoms Can Be Found for Tirant

The other three TEs that invaded *D. melanogaster* populations within the last 100 years (I-element, hobo, P-element) caused some hybrid dysgenesis (HD) symptoms. To test whether Tirant also induces HD symptoms, we performed crosses between strains having recent Tirant insertions (Urbana-S and Hikone-R) and strains not having such insertions (Lausanne-S and Canton-S). All strains do not have recent P-element, I-element, and hobo insertions, which rules out interference by the other HD systems (fig. 2*A* and supplementary table 1, Supplementary Material online). We investigated the fraction of dysgenic ovaries in the F1 generation, a trait influenced by P-element and hobo mobilization (Kidwell et al. 1977; Blackman et al. 1987; Yannopoulos et al. 1987), and the fraction of hatched F2 embryos, a trait influenced by I-element mobilization (Bucheton et al. 1976). We performed all crosses at several temperatures (supplementary fig. 17*A* and *B*, Supplementary Material online), as temperature frequently has a strong influence on the extent of HD symptoms (Kidwell et al. 1977; Bucheton 1979; Kidwell and Novy 1979; Serrato-Capuchina et al. 2020). We did not find any significant differences in the number of dysgenic ovaries nor in the number of hatched eggs between the reciprocal crosses (supplementary fig. 17*A* and B and table 5, Supplementary Material online). As the number of paternally inherited TEs may influence the magnitude of HD (Serrato-Capuchina et al. 2020), we performed reciprocal crosses with the strain carrying the largest number of canonical Tirant insertions, that is, Iso-1, and strains not having canonical Tirant insertions (Lausanne-S and Crimea; supplementary fig. 1, Supplementary Material online). However, Iso-1 also carries I-element and hobo insertions (supplementary fig. 1, Supplementary Material online). Therefore, we performed crosses solely at 25 °C, a temperature where I-element HD is usually not observed (Bucheton et al. 1976). As strains inducing hobo HD are very rare (Pascual and Periquet 1991), there is solely a small chance that hobo activity will generate atrophic ovaries in this crosses. We again did not find any significant differences in the number of dysgenic ovaries nor in the number of hatched eggs among these crosses (supplementary fig. 17*C* and D and table 5, Supplementary Material online; which also rules out hobo HD).

We hypothesize that the absence of apparent HD symptoms may be one reason why the invasion of Tirant in natural *D. melanogaster* populations during the past century was not detected before.

### Origin of the Canonical Tirant Invasion

Lastly, we aimed to shed light on the origin of the Tirant invasion. Since canonical Tirant insertions are mostly absent in strains collected before 1938, we reasoned that the recent Tirant invasion was likely triggered by HT (or an introgression). To identify the putative donor species, we investigated Tirant sequences in different *Drosophila* species. We first tested if Tirant sequences can be found in 11 sequenced *Drosophila* genomes (*Drosophila* 12 Genomes Consortium 2007). Solely members of the *D. melanogaster* species subgroup contained reads mapping to Tirant (supplementary fig. 18, Supplementary Material online; *D. melanogaster*, *D. simulans*, *D. erecta*, *D. yakuba*; in agreement with Fablet et al. [2007]). We also found that *D. simulans* is the only species that may carry full-length insertions of Tirant (apart from *D. melanogaster*) and that some Tirant insertions in *D. simulans* may have a high similarity to the consensus sequence of Tirant (supplementary fig. 18, Supplementary Material online). To further investigate the composition of Tirant in the *D. melanogaster* species subgroup, we obtained Illumina short-read data for several individuals from different species of this subgroup. In addition to *D. melanogaster*, *D. simulans*, *D. erecta*, and *D. yakuba*, we also obtained data for *D. sechellia*, *D. mauritiana*, and *D. teisseri* (supplementary table 6, Supplementary Material online). A PCA based on the allele frequencies of Tirant SNPs confirms that the Tirant composition of recently collected *D. melanogaster* strains (>1938) is most similar to *D. simulans* strains (supplementary fig. 19, Supplementary Material online). The high similarity of some Tirant sequences between *D. melanogaster* and *D. simulans* was noted before (Fablet et al. 2006; Lerat et al. 2011; Bargues and Lerat 2017). However, an analysis based on the allele frequencies confounds the two subfamilies of Tirant in these two species, for example, canonical Tirant insertions (Tirant-C in *D. simulans*) and degraded Tirant insertions (Tirant-S in *D. simulans*) (Fablet et al. 2006). Therefore, to further investigate whether some Tirant insertions of *D. simulans* could have triggered the canonical Tirant invasion in *D. melanogaster*, we analyzed the Tirant content in a recent long-read based assembly of *D. simulans* (strain w^*XD*^^1^; Chakraborty et al. 2020). Indeed, we found that *D. simulans* carries three full-length insertions that have a high similarity to the consensus sequence of Tirant (average divergence: 1.97%, 1.56%, 1.60%; supplementary table 7, Supplementary Material online). We concluded that HT from *D. simulans* may have triggered the invasion of the canonical Tirant in *D. melanogaster* populations.

## Discussion

We show that the retrotransposon Tirant invaded most natural *D. melanogaster* populations between 1930 and 1950, possibly following HT from *D. simulans*. Tirant is thus the fourth TE that invaded *D. melanogaster* in the last century. We also provide the first comprehensive timeline of the recent TE invasions in *D. melanogaster* populations that is based on a consistent approach (i.e., the same method and strains). The canonical Tirant invaded natural *D. melanogaster* populations first followed by the I-element, hobo, and finally by the P-element. All investigated strains, including those lacking canonical Tirant insertions, carry highly degraded Tirant fragments, which likely stem from an ancient Tirant invasion predating the split of the *D. melanogaster* species subgroup (Fablet et al. 2007; Lerat et al. 2011). We demonstrate that piRNAs derived from canonical and diverged Tirant insertions can be clearly distinguished and suggest that piRNAs derived from degraded Tirant copies, which were present in all investigated strains, were unable to prevent the invasion of the canonical Tirant. We show that founder effects may be important components of TE invasions that may lead to a heterogeneous TE composition among populations. Finally, we did not find apparent HD symptoms among reciprocal crosses of strains with and without canonical Tirant insertions.

Our conclusion that Tirant recently invaded *D. melanogaster* is mainly based on the absence of canonical Tirant sequences in most strains collected before 1938 and their presence in strains collected after 1938. As an alternative explanation, most strains collected before 1938 could have lost the canonical Tirant sequences. It was, for example, proposed that non-African *D. simulans* populations lost canonical Tirant sequences (Fablet et al. 2006). But this alternative explanation seems unlikely as it requires the independent loss of canonical Tirant sequences in strains collected before 1938 but not in any strain collected after 1938. The low population frequency of euchromatic Tirant insertions (see also Kofler, Nolte, et al. 2015) and the high sequence similarity between the left and the right LTR of Tirant insertions (Bowen and McDonald 2001; Bergman and Bensasson 2007) are also in agreement with our hypothesis of a recent Tirant invasion. Our hypothesis of the recent Tirant invasion is also consistent with the interpretation of the data for the I-element, P-element, and hobo, where the absence of the (canonical) TE in old strains combined with the presence in young strains was taken as evidence for recent invasions of these elements (Kidwell 1983; Daniels, Chovnick, et al. 1990; Daniels, Peterson, et al. 1990; Bucheton et al. 1992).

Our data suggest that Tirant was the first TE that invaded natural *D. melanogaster* populations in the last century. However, these results need to be interpreted with caution as 1) there is some uncertainty about the sampling time of the strains, 2) some strains may have been contaminated (e.g., the presence of the P-element in a strain collected around 1938 [Swedish-C] is likely due to mixing of strains during maintenance of stocks; supplementary table 1, Supplementary Material online), and 3) our strains are from different geographic regions, where some regions might have been invaded earlier than others. Nevertheless, our results are largely in agreement with previous works which suggested that the I-element invasion happened between 1930 and 1950, the hobo invasion around 1955 and the P-element invasion between 1950 and 1980 (Kidwell 1983; Anxolabéhère et al. 1988; Periquet et al. 1989).

We did not find evidence that Tirant induces HD symptoms. Also, a previous work in *D. simulans* did not report HD symptoms for Tirant despite Tirant being activated by reciprocal crosses (Akkouche et al. 2013). However, due to several reasons, more work will be necessary to show whether or not Tirant causes some HD symptoms. First, it is not clear what symptoms to look for. We investigated the fraction of dysgenic ovaries in the F1 and the fraction of hatched eggs (F2), two traits affected by HD from P-element, I-element, or hobo. However, it is feasible that Tirant activity leads to entirely different phenotypic effects, especially given that Tirant may be active in the germline and in the soma (Malone et al. 2009; Akkouche et al. 2013; Czech et al. 2013), and could thus affect both tissues. Second, it is not clear if intermediate TEs, such as Tirant, are able to induce HD. Different phenotypes among reciprocal crosses (i.e., HD) can solely be observed if maternally transmitted piRNAs (i.e., the cytoplasmic component of HD) are necessary to silence a TE (Brennecke et al. 2008). Maternally transmitted piRNAs initiate the ping-pong cycle and recruit silencing chromatin that is then bound by Rhino, which in turn defines the site of dual-strand clusters (Le Thomas et al. 2014). As both ping-pong and dual-strand clusters are solely observed in the germline piRNA pathway (Malone et al. 2009), it is thought that maternally deposited piRNAs are important for the germline pathway but not for the somatic piRNA pathway. Consequently, no HD symptoms are expected for TEs that are solely active in the soma. The I-element, hobo, and the P-element, three TEs that invaded *D. melanogaster* populations recently, were all active in the germline and induced HD symptoms (Bingham et al. 1982; Calvi and Gelbart 1994; Biémont 2010; Moon et al. 2018; Wang et al. 2018). However, the situation is entirely unclear for intermediate elements such as Tirant. Surprisingly, one study even suggested that maternally transmitted piRNAs are necessary to silence Tirant in the soma (Akkouche et al. 2013). The molecular mechanisms behind this influence of maternal piRNAs on the somatic piRNA pathway remain yet unclear. Third, the severity of HD symptoms frequently depends on multiple factors, such as temperature and the age of flies (Kidwell et al. 1977; Bucheton 1979; Kidwell and Novy 1979; Serrato-Capuchina et al. 2020). It is feasible that HD symptoms of Tirant can only be observed under certain conditions, and these conditions could differ substantially from the previously described HD systems. Fourth, previous studies noted marked differences in the ability to induce or repress HD among different strains (Kidwell et al. 1977, 1988; Anxolabéhère et al. 1988; Pascual and Periquet 1991; Srivastav et al. 2019). This could be mediated by differences in the number of paternally transmitted TEs (Srivastav and Kelleher 2017; Serrato-Capuchina et al. 2020), different variants of the TEs (Srivastav et al. 2019), and differences in the tolerance to TE activity among strains (Kelleher et al. 2018). The abundance of strains inducing HD may also vary among the HD systems. For example, strains inducing P-element HD are readily found whereas strains inducing hobo HD are rare (Kidwell 1983; Pascual and Periquet 1991). It is thus feasible that solely crosses of certain strains show HD symptoms of Tirant.

It is currently unclear how canonical Tirant sequences entered *D. melanogaster* populations. Possible explanations are HT or introgression from a related species (Silva et al. 2004; Sánchez-Gracia et al. 2005; Loreto et al. 2008; Bartolomé et al. 2009). In search for a possible donor species, we found that *D. simulans* carries some full-length Tirant insertions with a high similarity to canonical Tirant in *D. melanogaster* (supplementary table 7, Supplementary Material online). Out of the two Tirant subfamilies found in *D. simulans*, Tirant-C (nondegraded insertions) and Tirant-S (degraded insertions), Tirant-C insertions have been previously shown to be closely related to the canonical Tirant in *D. melanogaster* (Fablet et al. 2006; Lerat et al. 2011; Bargues and Lerat 2017). We thus suggest that HT of Tirant-C from *D. simulans* to *D. melanogaster* may have triggered the canonical Tirant invasion in *D. melanogaster*, in agreement with Lerat et al. (2011). Apart from this HT, Tirant is likely mostly vertically transmitted in the *D. melanogaster* species subgroup (Fablet et al. 2007). In agreement with this, a tree based on frequency of Tirant SNPs largely follows the species tree (supplementary fig. 20, Supplementary Material online). HT of TEs between *D. melanogaster* and *D. simulans* is plausible since both species are closely related (Lemeunier et al. 1976) and have largely overlapping habitats (Parsons and Stanley 1981), which generates ample opportunities for HT or introgressions. HT of TEs between these species was observed before in both directions. For example, Kofler, Hill, et al. (2015) suggested that *D. simulans* recently acquired the P-element from *D. melanogaster*. Conversely, hobo and the I-element in *D. melanogaster* were possibly acquired from *D. simulans* (Daniels, Chovnick, et al. 1990; Simmons 1992; Loreto et al. 2008).

We found that Tirant sequences from Tasmania (an island south of Australia) have a different composition than Tirant sequences from other locations (at least five SNPs have distinctly different frequencies; supplementary table 4, Supplementary Material online). We suggest that this may be due to a founder effect during the Tirant invasion, which led to the spread of rare Tirant variants in Tasmanian populations. We wondered whether the observed founder effect could be due to the recent colonization of Australia (Tasmania) by *D. melanogaster* (Bock and Parsons 1981). However, this seems unlikely as the colonization of Australia, and probably also of Tasmania, predates the Tirant invasion. *Drosophila melanogaster* was first spotted in Australia in 1894 and is known to rapidly spread into nearby areas (Bock and Parsons 1981; Keller 2007), whereas the Tirant invasion mostly happened between 1938 and 1950. Moreover, founder effects that occurred during the colonization of Tasmania should affect the entire genomic background of *D. melanogaster* and not just the Tirant sequences. Previous studies did not detect any signatures of bottlenecks for Tasmanian *D. melanogaster* populations (Agis and Schlötterer 2001; Grenier et al. 2015; Bergland et al. 2016; Arguello et al. 2019). We thus argue that the founder effect in Tasmania is specific to Tirant. Founder effects during TE invasions could be important, hitherto little considered, processes that may lead to geographically distinct TE variants.

We suggest that four different TEs invaded *D. melanogaster* populations within 40 years (between the 1930s and 1970s). Why did so many different TEs spread in *D. melanogaster* within such a short time? A possible explanation could be the recent habitat expansion of *D. melanogaster* into the Americas and Australia about 100–200 years ago (Bock and Parsons 1981; Vieira et al. 1999; Kofler, Nolte, et al. 2015). Habitat expansion may bring species into contact that did not coexist before in the same habitat. If these species carry different TE families, HT events between the species may trigger novel TE invasions. A classic example is the P-element in *D. melanogaster* which was likely acquired from *D. willistoni* after *D. melanogaster* entered the habitat of *D. willistoni* in South America (Engels 1992). The lag-time between colonization of the Americas and Australia (∼100–200 years ago; Bock and Parsons 1981; Keller 2007) and the four different TE invasions (1930–1970) may be due to the stochasticity of HT events, a strong influence of drift in the early stages of TE invasions and the time required until a TE reaches an appreciable frequency (Ginzburg et al. 1984; Le Rouzic and Capy 2005). It will be interesting to see if such a high rate of novel TE invasions in *D. melanogaster* populations will be maintained over the next century. An absence of novel invasions would support our hypothesis that the habitat expansion triggered the four recent TE invasions in *D. melanogaster*.

Out of the four TEs that invaded *D. melanogaster* populations in the last century, the P-element is unique as it is the only TE that does not show substantial similarity to any sequence of the *D. melanogaster* genome. For the other three TEs—Tirant, the I-element, and hobo—many degraded insertions can be found (mostly in the heterochromatin) (Bucheton et al. 1984, 1986, 1992; Daniels, Chovnick, et al. 1990). Thus, three out of the four TEs probably invaded *D. melanogaster* populations at least twice. This raises the question of how multiple waves of invasions arise. Before a TE can trigger a novel invasion the TE needs to overcome the host defense (or the host defense may break down). For example, in mammals and invertebrates efficient silencing of a TE requires piRNAs that match with less than 10% sequence divergence over the bulk of the TE sequence (Post et al. 2014; Kotov et al. 2019). A TE that diverged by more than 10% from the piRNA pool of the host (e.g., the canonical Tirant compared with the degraded Tirant sequences) could thus trigger a second wave of an invasion. The same consideration holds for other host defense mechanism that rely on sequence similarity to a TE, like small RNAs in plants or Kruppel-associated box zinc-finger proteins in mammals (Marí-Ordóñez et al. 2013; Yang et al. 2017). It is however an important open question whether sufficient sequence divergence could be acquired within a host species, where host defense mechanisms may coadapt with the TE, or whether HT to an intermediate host (e.g., a closely related species) is necessary to overcome the host defense.

## Materials and Methods

### Strains and Dating

The sequenced fly strains were obtained from the Bloomington Drosophila Stock Center (BDSC) (Crimea, Lausanne-S, Swedish-C, Urbana-S, Berlin-K, Hikone-R, Florida-9, Pi2, Harwich, Amherst-3) and the National Drosophila Species Stock Center (Dmel68). w1118 and wk were kindly provided by Silke Jensen. We additionally analyzed publicly available sequencing data of different *D. melanogaster* strains (King et al. 2012; Mackay et al. 2012; Bergland et al. 2014; Grenier et al. 2015; Lack et al. 2015; Jakšić et al. 2017; Machado et al. 2019; Kapun et al. 2020; Wierzbicki et al. 2020) (supplementary table 6, Supplementary Material online). The collection dates of the strains were obtained from different sources. If available, we used the collection dates from Lindsley and Grell (1968). Alternatively, we used the collection dates published in previous works (Black et al. 1987; Anxolabéhère et al. 1988; Galindo et al. 1995; Engels 2007; Ruebenbauer et al. 2008) or information from the National Drosophila Species Stock Center (drosophilaspecies.com) and FlyBase (flybase.org/reports/FBrf0222222.html, last accesssed December 15, 2020) (supplementary table 1, Supplementary Material online). For the strains w1118 and Urbana-S, we used the latest possible collection date: for w1118, we used the publication date of the first publication mentioning the strain and for Urbana-S, we used the year of the death of C. Bridges, who collected the strain (Lindsley and Grell 1968) (supplementary table 1, Supplementary Material online). The geographic origin was obtained from the same sources. For an overview of the used strains, the estimated collection date, and the source of the information, see supplementary table 1, Supplementary Material online. The Iso-1 strain was generated by crossing several laboratory strains, with largely unknown sampling dates (Brizuela et al. 1994). Therefore, we did not assign a sampling date to this strain. Additionally, we used publicly available data of different strains from *D. simulans*, *D. sechellia*, *D. mauritiana*, *D. yakuba*, *D. teisseri*, and *D. erecta* (*Drosophila* 12 Genomes Consortium 2007; Garrigan et al. 2012, 2014; Rogers et al. 2014; Turissini et al. 2015; Melvin et al. 2018; Miller et al. 2018; Schrider et al. 2018; Cooper et al. 2019; Kang et al. 2019; Lanno et al. 2019; Meany et al. 2019; Stewart and Rogers 2019). For an overview of all used publicly available data, see supplementary table 6, Supplementary Material online.

### DNA Sequencing

DNA for Illumina paired-end sequencing was extracted from whole bodies of 20–30 virgin female flies using a salt-extraction protocol (Maniatis et al. 1982). Libraries were prepared with the NEBNext Ultra II DNA library Prep Kit (New England Bioloabs, Ipswich, MA) using 1 μg DNA. Illumina sequencing was performed by the Vienna Biocenter Core Facilities using the HiSeq2500 platform (2 × 125 bp; Illumina, San Diego, CA).

### Small RNA Sequencing

For small RNA sequencing, we extracted total RNA from ovaries of the strains Canton-S, Iso-1, and Lausanne-S using TRIzol. The small RNA was sequenced by Fasteris (Geneva, Switzerland). After depletion of 2S rRNA, library preparation was performed using the Illumina TruSeq small RNA kit and cDNA was sequenced on an Illumina NextSeq platform (50 bp; Illumina, San Diego, CA). Adapter sequences were trimmed with cutadapt (v2.3) (Martin 2011) (adapter: TGGAATTCTCGGGTGCCAAGGAACTCCAGTCACCATTTTATCTCGTATGC) and filtered for reads with a length between 18 and 36 nt. The reads were mapped to a database consisting of *D. melanogaster* miRNAs, mRNAs, rRNAs, snRNAs, snoRNAs, tRNAs (Thurmond et al. 2019), and the TE sequences (Quesneville et al. 2005) using novoalign (v3.09; http://novocraft.com/, last accesssed December 15, 2020) and allowing for two mismatches (unless mentioned otherwise). Solely piRNAs with a length between 23 and 29 nt were retained and the abundance of piRNAs was normalized to a million miRNAs as described previously (Kofler et al. 2018). For computing the ping-pong signatures and visualizing the piRNA abundance along the Tirant sequence, we used a previously developed pipeline (Kofler et al. 2018). To calculate the abundance of piRNAs complementary to the degraded Tirant fragments, we first extracted the sequences of degraded Tirant insertions (>10% divergence to consensus sequence) from the reference assembly of Iso-1 (v6.22) with RepeatMasker (open-4.0.7; Smit et al. 2013–2015) and bedtools (Quinlan and Hall 2010) (v2.29.2). All sequences longer than 100 bp were concatenated (the reverse complement was adjusted with bedtools) and small RNAs were mapped to these sequences using novoalign. The abundance of all piRNAs complementary to degraded Tirant sequences was summed. We also analyzed the small RNA content of the five GDL strains B10, I06, N10, T05, and ZW155 (data are publicly available; Luo et al. 2020).

### TE Abundance and Diversity

The coverage along a TE and the frequencies of SNPs and indels in a TE were computed using our newly developed tool DeviaTE (v0.3.8) (Weilguny and Kofler 2019). Briefly, short reads from a sample were aligned with bwa sw (v0.7.17) (Li and Durbin 2009) to the TE consensus sequences of *Drosophila* (Quesneville et al. 2005) as well as to three single-copy genes (*traffic jam*, *rpl32*, and *rhino*), which allowed us to infer TE copy numbers by contrasting the coverage of a TE to the coverage of the single-copy genes. The abundance and diversity of TE insertions were visualized with DeviaTE (Weilguny and Kofler 2019). To obtain the normalized number of reads mapping to each TE (rpm), we used PopoolationTE2 (v1.10.03) (Kofler et al. 2016). Based on the visualization of the TE composition with DeviaTE and the estimates of the TE abundance (rpm and DeviaTE using normalization with single-copy genes), we manually classified the presence/absence of Tirant, hobo, the I-element, and the P-element in different *D. melanogaster* strains (supplementary table 1, Supplementary Material online). We used the following three categories: 1) absence of any TE sequences, 2) solely degraded TE sequences are present, 3) nondegraded sequences, with a high similarity to the consensus sequence, are present. For example, see supplementary figures 4 and 6–8, Supplementary Material online. A PCA based on the allele frequencies of SNPs in a TE supports our classification for Tirant and hobo. Since many strains do not contain any P-element sequences, the allele frequencies of SNPs in the P-element could not be calculated for all strains. Despite discernible differences between strains with and without recent I-element insertions, the PCA did not separate these two groups (supplementary figs. 6 and 14, Supplementary Material online). The PCA was performed in R (prcomp) using arcsine and square root transformed allele frequencies of SNPs in TEs (R Core Team 2012). The DSPR lines were not included into the PCA due to their short-read length (50 bp). The pairwise *F*_ST_ based on the SNPs of TEs was computed with Popoolation2 (v1.2.01) (Kofler et al. 2011) (“fst-sliding.pl” –window-size 8526 –max-coverage 0.1%).

We used PoMo (Schrempf et al. 2016) based on the allele frequencies of Tirant SNPs to generate a tree of the species in the *D. melanogaster* species subgroup. PoMo uses allele frequency data to account for the intraspecific differences while calculating the interspecific variation. We run PoMo with IQ-TREE (v1.6.12) (Nguyen et al. 2015) using polymorphism-aware models (HKY + P). We obtained bootstrap estimates for each node using the ultra-fast bootstrap (-bb) option for 1000 replicates.

Tirant sequences in the assemblies of Canton-S (Wierzbicki et al. 2020) and Iso-1 (v6.22; https://flybase.org/, last accessed December 15, 2020) were identified with RepeatMasker using the TE consensus sequences of *Drosophila* as custom library (Quesneville et al. 2005). To visualize the divergence of annotated Tirant fragments of the Canton-S genome, we extract all sequences annotated with RepeatMasker and map them to the Tirant consensus sequence using bwa sw (Li and Durbin 2009) with a low mismatch penalty (-b) of 0.5. Visualization of the sequence alignment was done with IGV. Colored lines represent SNPs compared with the consensus sequence.

We searched for canonical Tirant insertions in a long-read based assembly of *D. simulans* (strain w^*XD*^^1^; PRJNA383250; Chakraborty et al. 2020) using RepeatMasker (open-4.0.7; Smit et al. 2013–2015). We filtered for complete insertions with a low divergence (<5%).

To estimate the position and population frequency of canonical and degraded Tirant insertions in a natural *D. melanogaster* population, we used PoPoolationTE2 (v1.10.03) (Kofler et al. 2016) and a population collected in 2014 at Viltain (France) by the DrosEU consortium (SRR5647729; Kapun et al. 2020). We generated the artificial reference genome required by PoPoolationTE2, by merging the repeat masked reference genome, the consensus sequence of Tirant and the degraded Tirant sequences with a minimum length of 100 bp (see above) into a single fasta file. The short reads were mapped to this artificial reference genome using bwa mem (v0.7.17) (Li and Durbin 2009) with paired-end mode and the parameter -M. The mapped reads were sorted with samtools (Li, Handsaker, et al. 2009). Finally, we followed the PoPoolationTE2 pipeline using the parameters: –map-qual 15, –min-count 2, –min-coverage 2. We indicated heterochromatic regions following previous work (Riddle et al. 2011; Hoskins et al. 2015).

### Hybrid Dysgenesis Assay

To test whether Tirant induces HD symptoms, we performed four reciprocal crosses among *D. melanogaster* strains having canonical Tirant insertions (Urbana-S, Hikone-R, and Iso-1) and strains not having canonical Tirant insertions (Lausanne-S, Canton-S, Crimea). Each cross was performed in three replicates by mating 20 female virgin flies with 15 males. To estimate the number of dysgenic ovaries, 2–5 days old F1 flies (kept at either 20, 25, or 29 °C) were allowed to lay eggs on black agar plates (containing charcoal) for 24 h. The F1 female ovaries were dissected on PBS and scored for the presence of dysgenic (underdeveloped) ovaries. The deposited F2 embryos were counted, incubated for 24 h, and the number of larvae (=hatched eggs) was quantified. Crosses involving Iso-1 were only performed at 25 °C.

## Supplementary Material

Supplementary data are available at *Molecular Biology and Evolution* online.

## Supplementary Material

msaa308_Supplementary_Data
