# Supplementary material for: Tirant Stealthily Invaded Natural Drosophila melanogaster Populations during the Last Century
Source: Mol Biol Evol. 2020 Nov 28;38(4):1482–97. doi: 10.1093/molbev/msaa308 (PMC8042734; doi:10.1093/molbev/msaa308)
Supplement: msaa308_Supplementary_Data [file msaa308_supplementary_data.zip › supplement_revised.pdf]

Supplement to Tirant stealthily invaded natural *Drosophila melanogaster* populations during the last century

Florian Schwarz, Filip Wierzbicki, Kirsten-André Senti and Robert Kofler

October 29, 2020

Supplementary figures

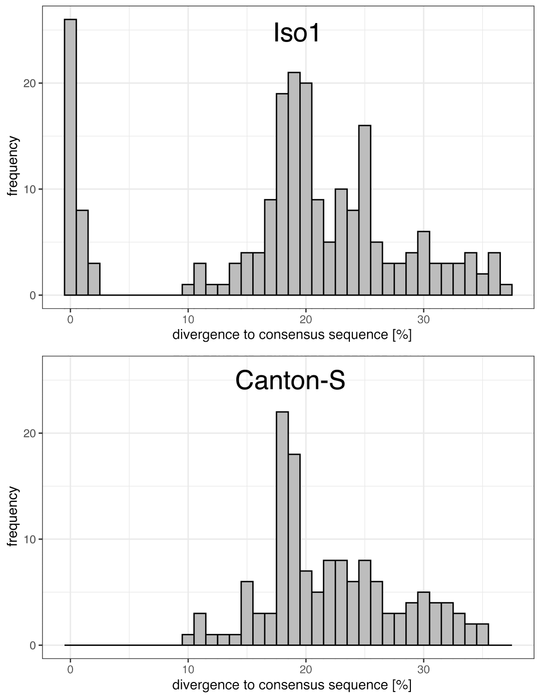

Figure 1: Histogram of the divergence of Tirant sequences relative to the canonical sequence. Tirant sequences were annotated with RepeatMasker in the assemblies of the *D. melanogaster* strains Iso-1 and Canton-S. Note that Iso-1 contains canonical (divergence < 5%) as well as degraded (divergence > 10%) Tirant insertions, whereas Canton-S solely contains degraded Tirant insertions.

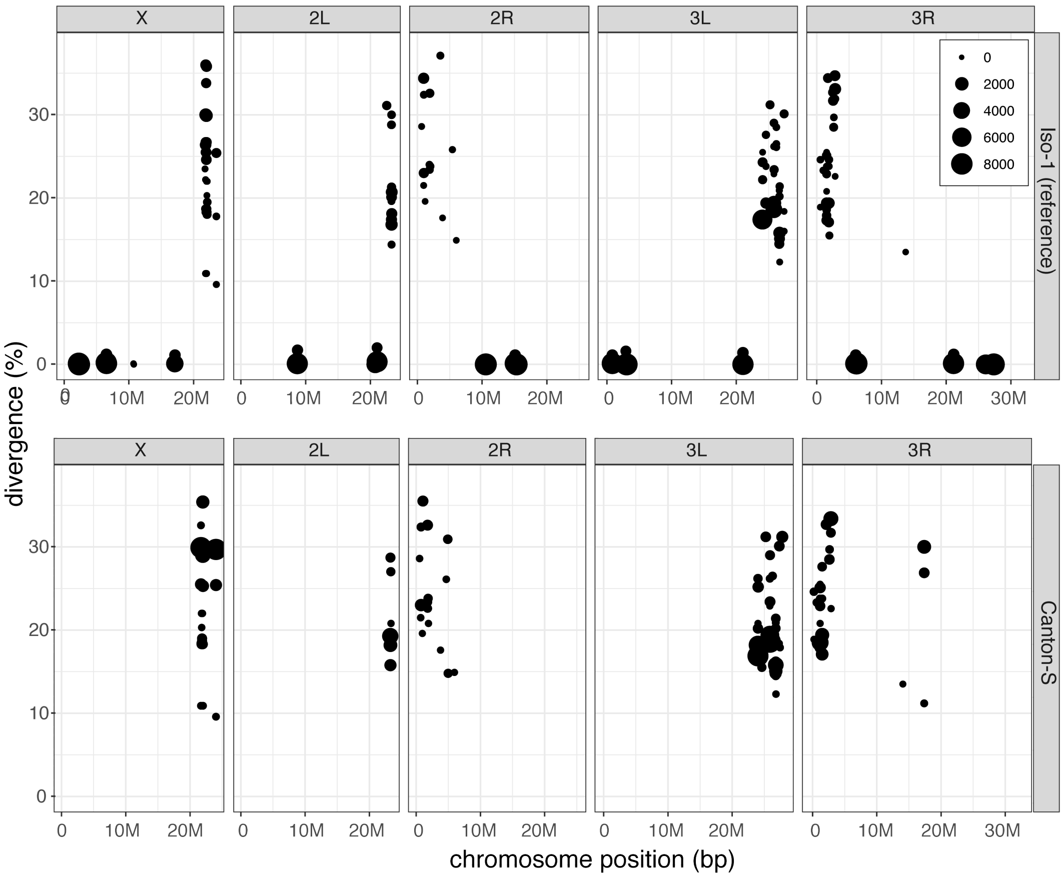

Figure 2: Canonical Tirant insertions are present in Iso-1 but not in Canton-S. The Canton-S assembly was generated by Chakraborty et al. (2019) with PacBio reads (the Canton-S assembly shown in the main manuscript was generated by Wierzbicki et al. (2020) with ONT reads). For each Tirant insertion we show the position in the assembly, the length (size of dot), and the similarity to the consensus sequence (divergence).

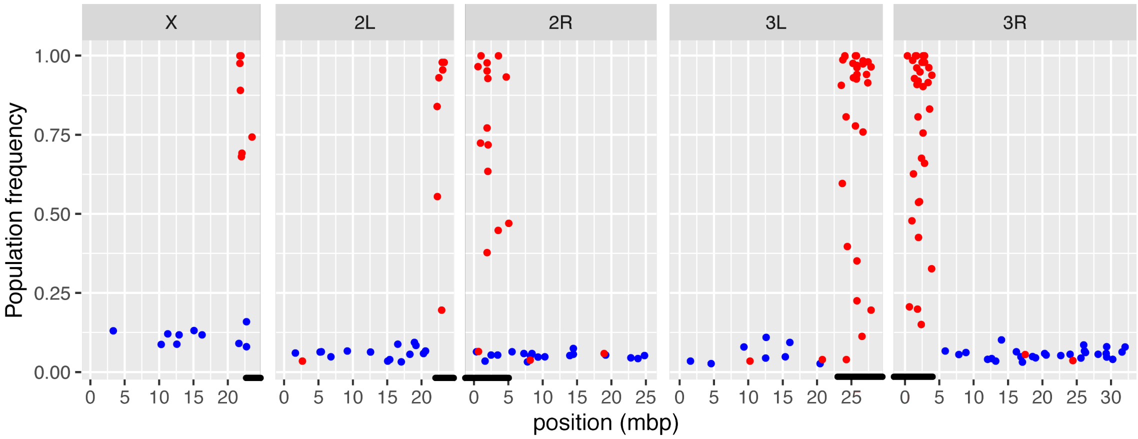

Figure 3: Position and population frequency of canonical (blue) and degraded (red) Tirant insertions in a population from France (Viltain) (Kapun et al., 2020). Canonical Tirant insertions are mostly euchromatic and segregating at a low population frequency whereas degraded insertions are mostly heterochromatic and segregating at high frequency. Black bars indicate (peri)centric heterochromatin (Riddle et al., 2011; Hoskins et al., 2015).

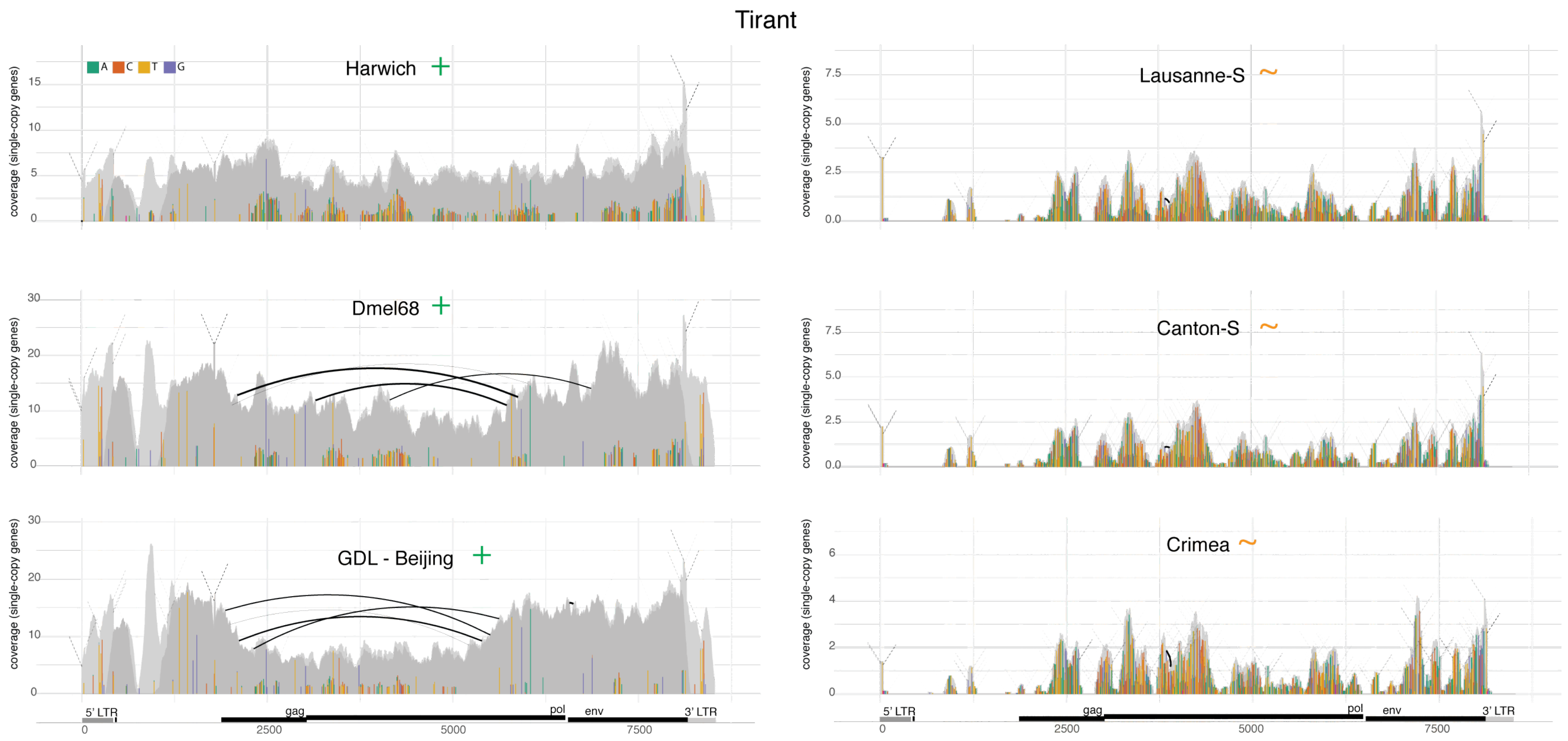

Figure 4: DeviaTE plots for three strains having non-degraded (i.e. canonical) Tirant sequences (+) and three strains solely having degraded Tirant sequences (~). Such plots were used for classifying the Tirant content of the different *D. melanogaster* strains (see supplementary table 1).

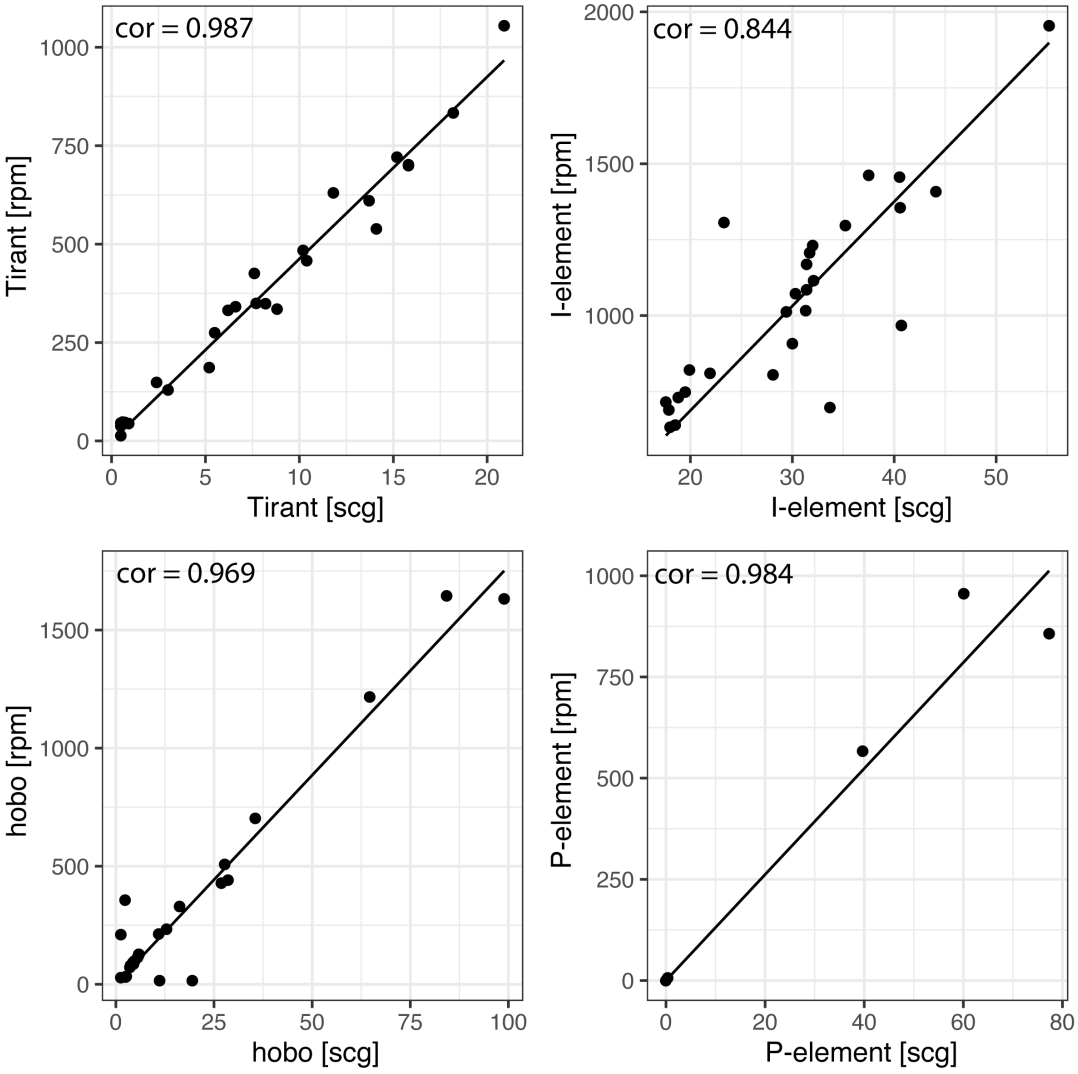

Figure 5: Correlation between the TE abundance estimated by DeviaTE using single copy gene normalization (scg) and the raw abundance of reads mapping to each TE normalized to a million reads (rpm: reads per million). Data are reported for Tirant, the I-element, hobo and the P-element. cor, Pearson's correlation coefficient

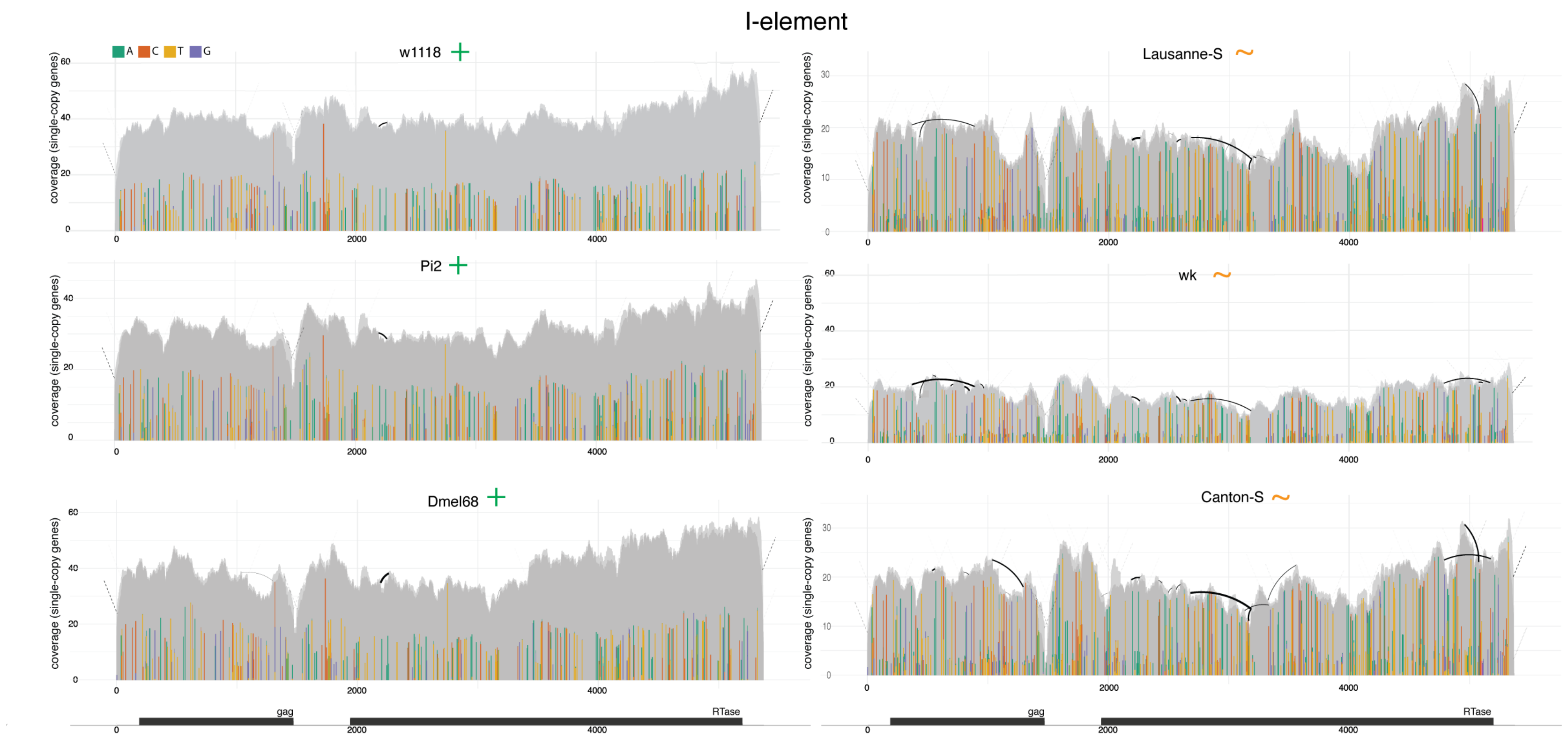

Figure 6: DeviaTE plots for three strains having non-degraded I-element sequences (+) and three strains solely having degraded I-element sequences (~). Such plots were used for classifying the I-element content of the different *D. melanogaster* strains (see supplementary table 1).

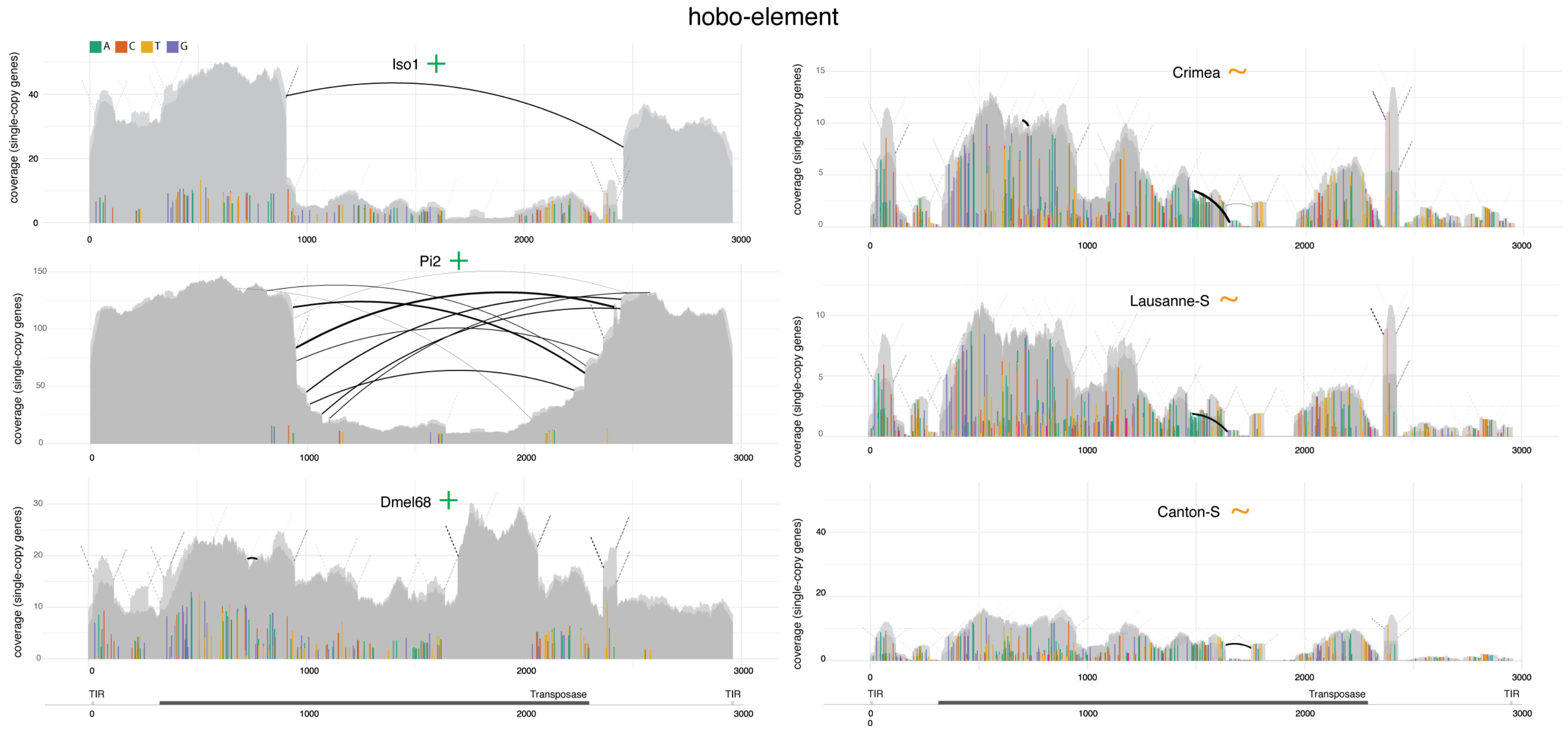

Figure 7: DeviaTE plots for three strains having non-degraded hobo sequences (+) and three strains solely having degraded hobo sequences (~). Such plots were used for classifying the hobo content of the different *D. melanogaster* strains (see supplementary table 1).

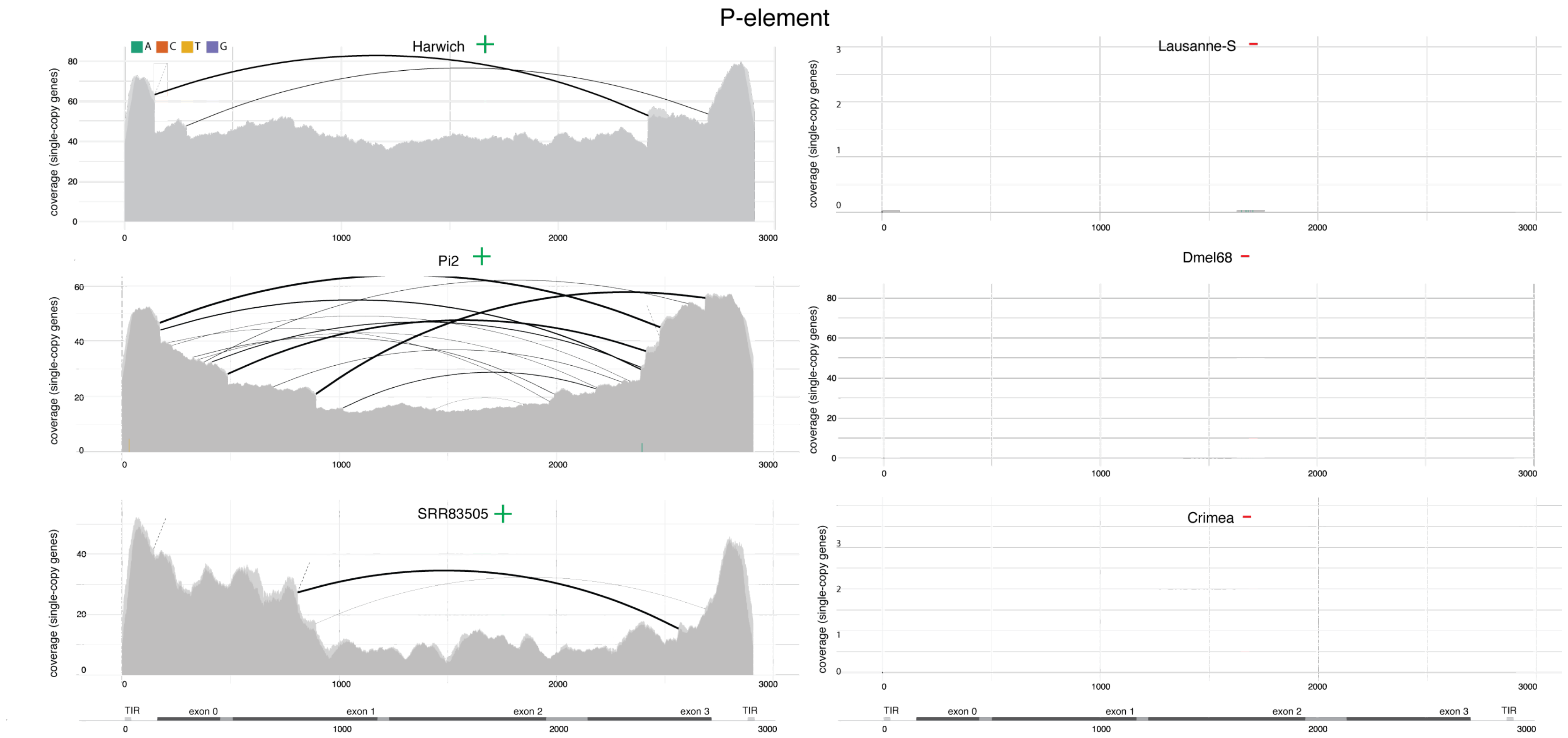

Figure 8: DeviaTE plots for three strains having P-element sequences (+) and three strains not having P-element sequences (−). Such plots were used for classifying the P-element content of the different *D. melanogaster* strains (see supplementary table 1).

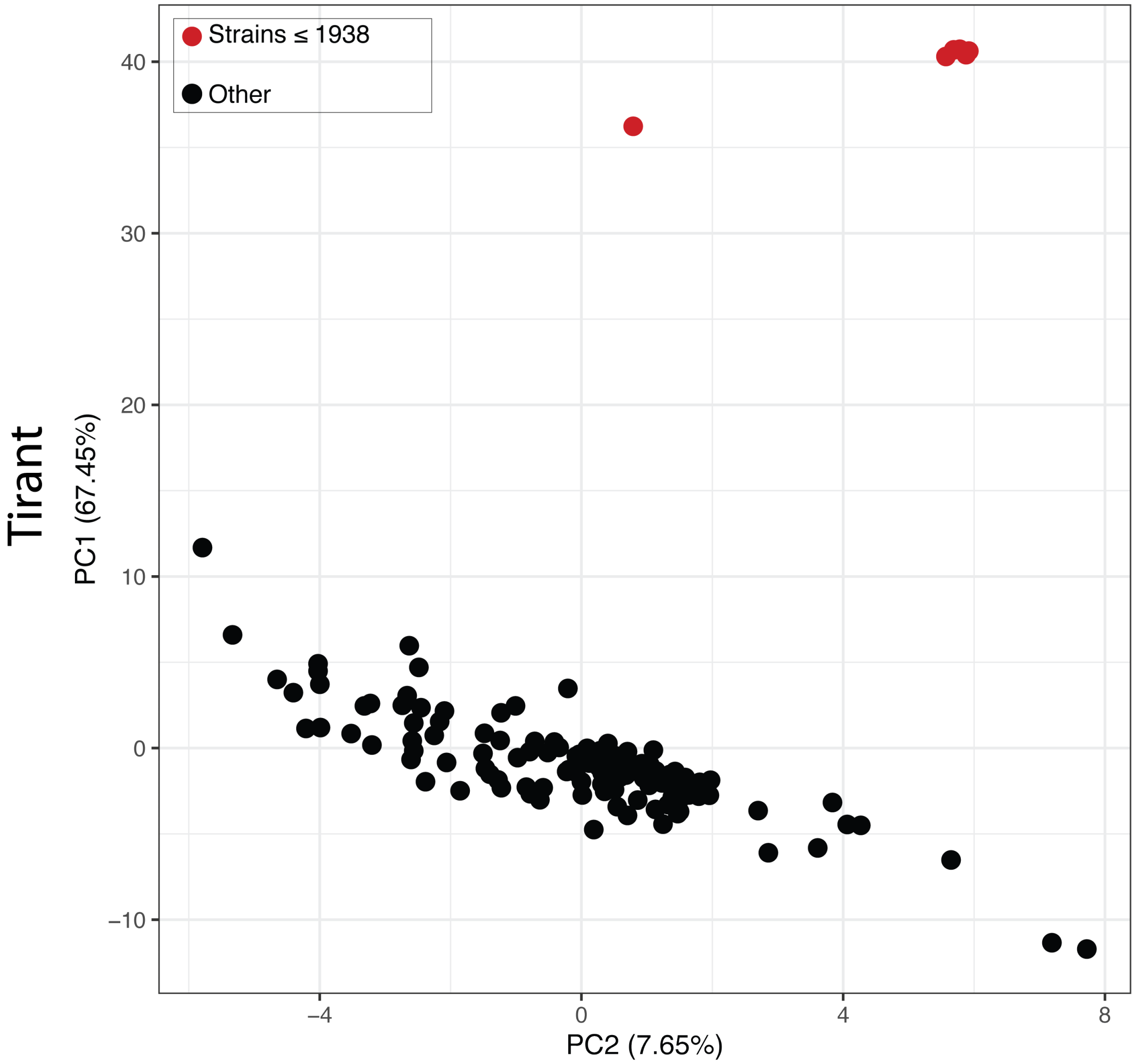

Figure 9: PCA based on the allele frequencies of SNPs in Tirant for different *D. melanogaster* strains and population samples. Strains sampled before or at 1938 form a separate cluster (due to the absence of canonical Tirant insertions). In addition to the strains shown in the manuscript (fig. 3), we used DGRP, DrosEU and Dros-RTEC lines well as lines sampled by Bergland et al. (2014) and Lack et al. (2015) (see supplementary table 1 for details).

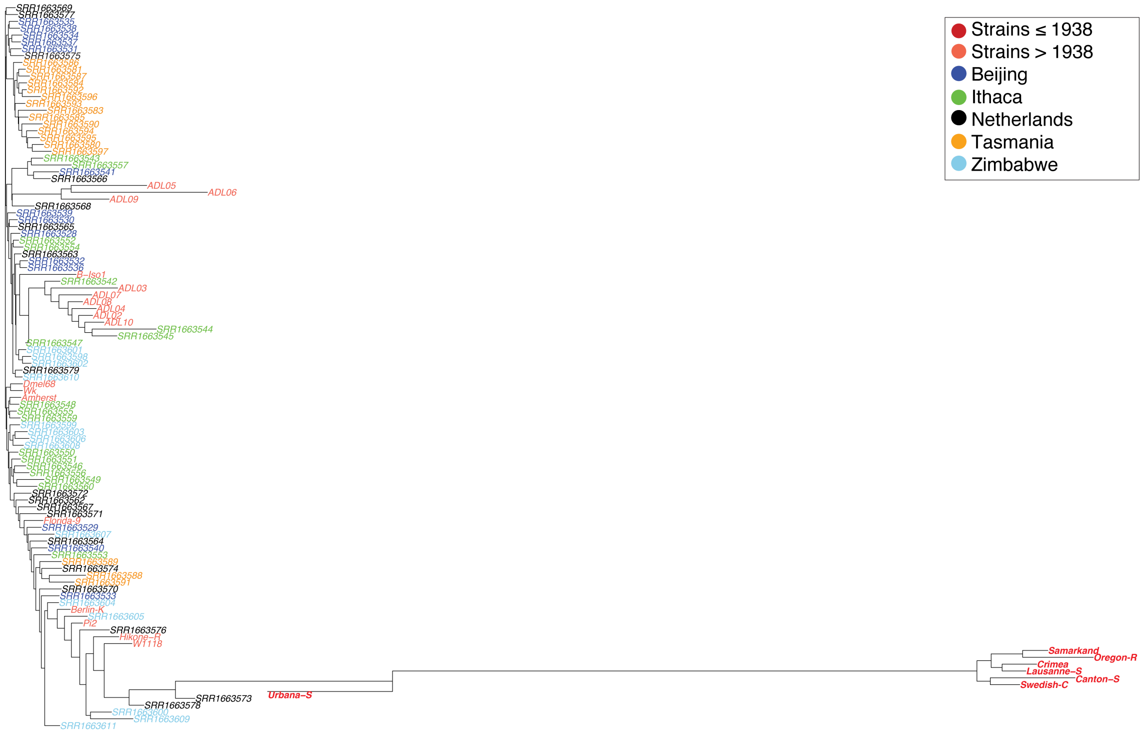

Figure 10: Unrooted tree detailing similarity in the Tirant composition among different *D. melanogaster* strains and population samples. The tree is based on a pairwise distance matrix of F_{ST} values computed from the allele frequencies of Tirant SNPs. Names of strains are colored by spatial or temporal origin. Old strains (< 1938) are additionally shown in bold. Note that old strains (< 1938) and most strains from Tasmania form distinct groups.

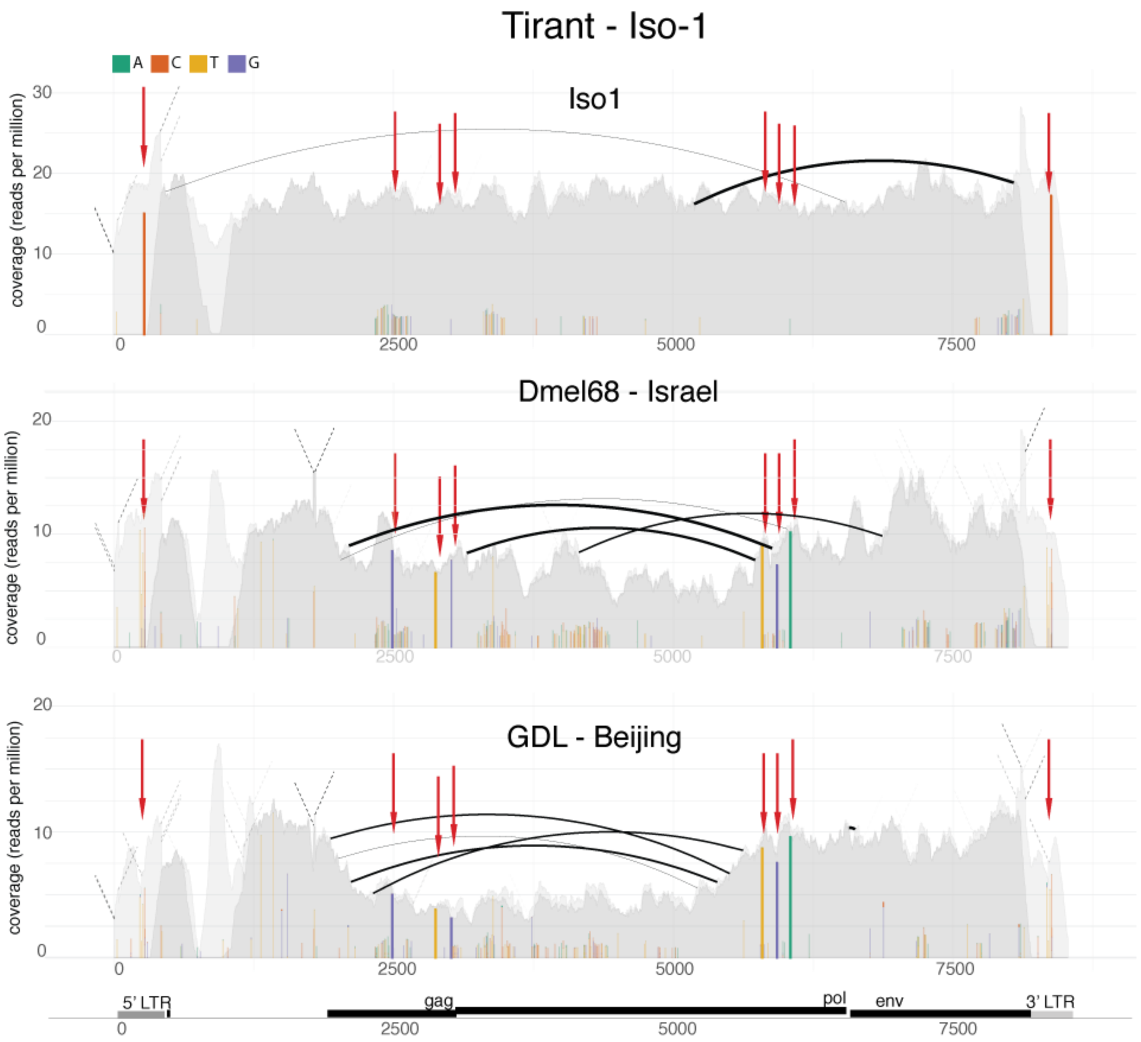

Figure 11: Abundance and diversity of Tirant in the reference strain Iso-1 and two strains collected from natural *D. melanogaster* populations (Dmel68 and a GDL line from Beijing). Eight SNPs found in natural populations but not in Iso-1 are marked by red arrows. To enhance the visibility of these SNPs, opacity of the background was reduced and the size of the SNPs was increased.

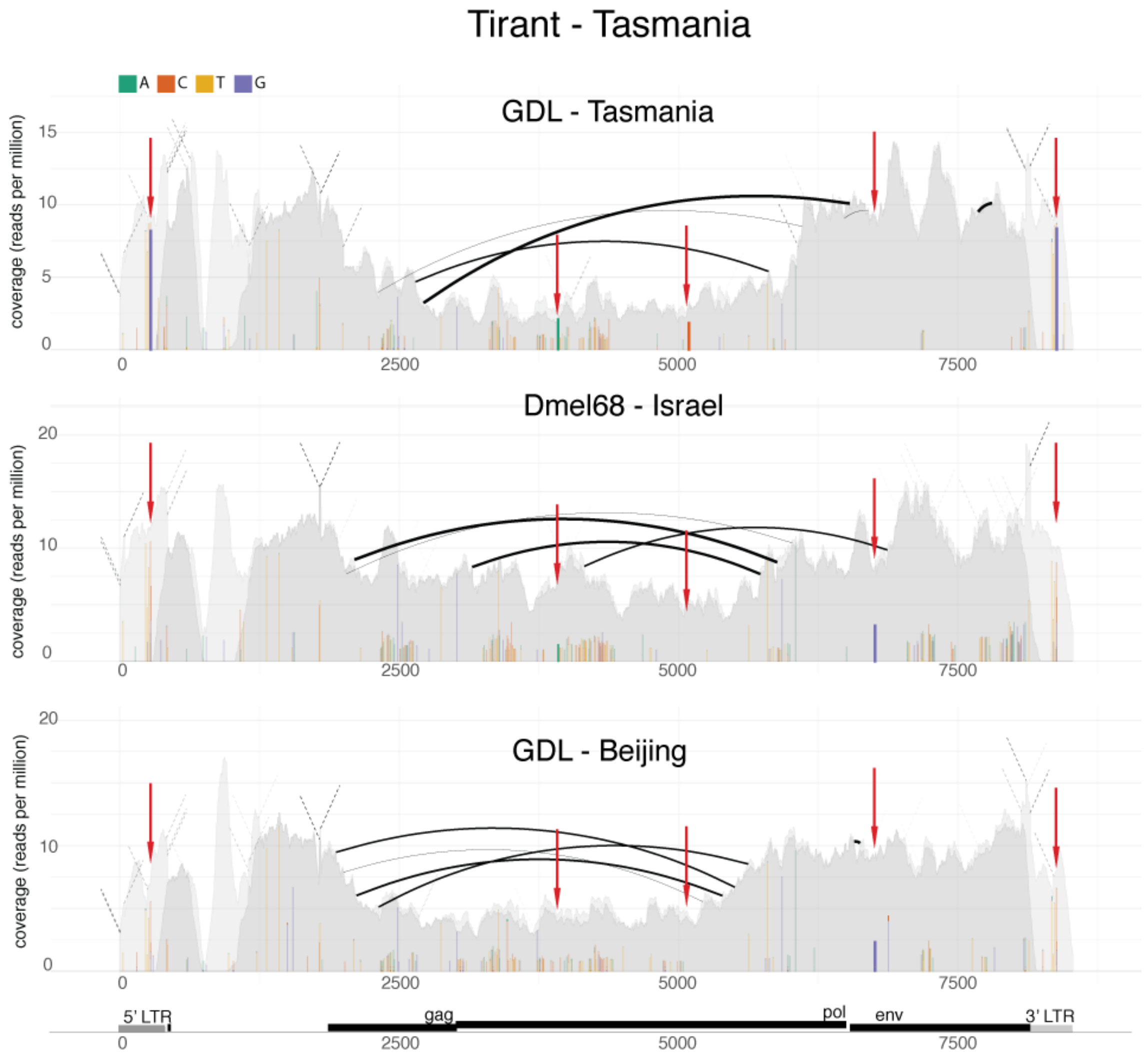

Figure 12: Abundance and diversity of Tirant sequences in a natural population from Tasmania and from other geographic locations. Five SNPs, marked by red arrows, have notably different allele frequencies between populations from Tasmania and the other geographic locations. To enhance the visibility of these SNPs, opacity of the background was reduced and the size of the SNPs was increased.

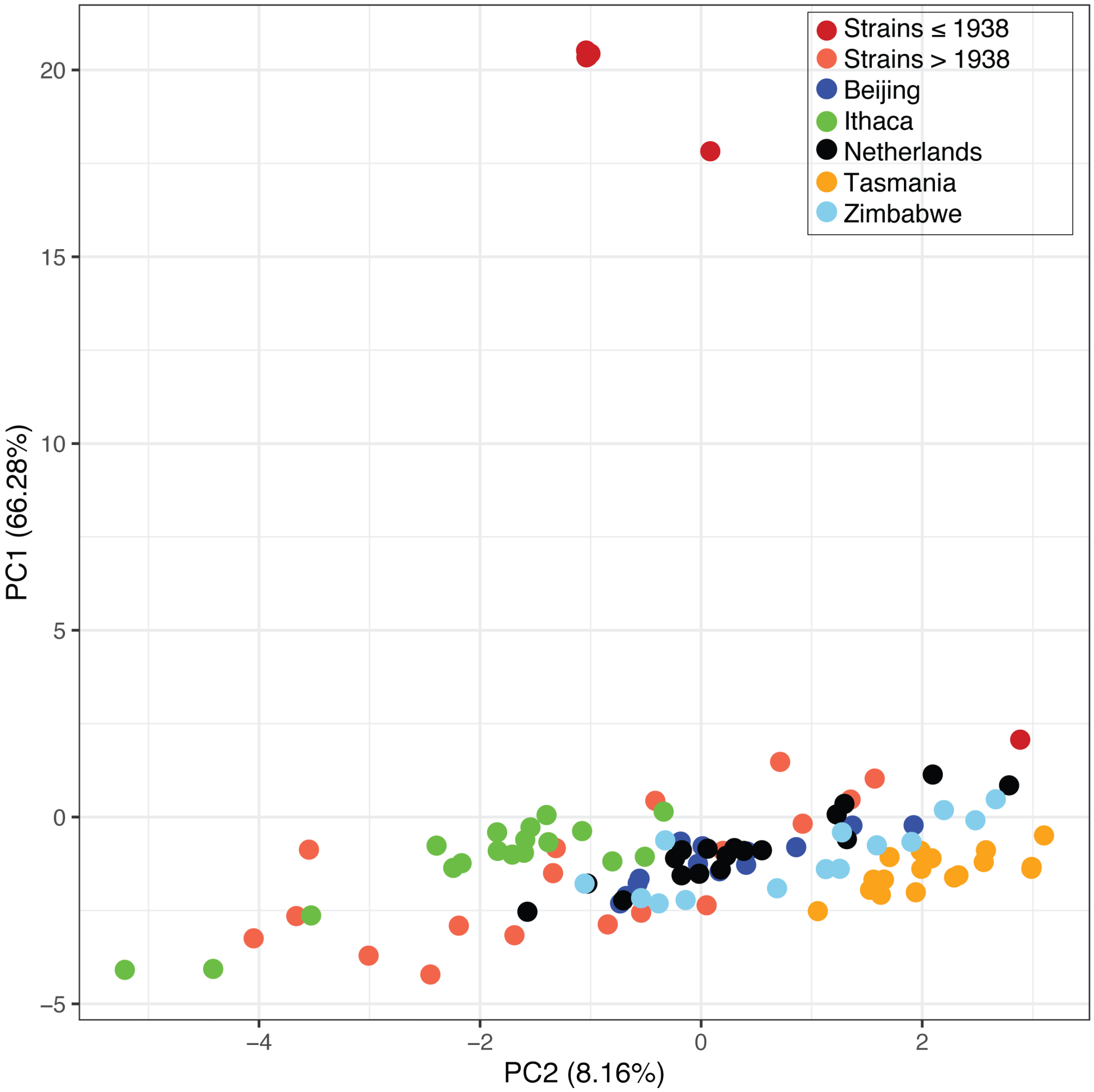

Figure 13: PCA based on the allele frequencies of SNPs in Tirant for different *D. melanogaster* strains and population samples as displayed in Figure 2. The five variants described in supplementary table 4 were omitted from the analysis, which removes the distinct clustering of the Tasmanian strains.

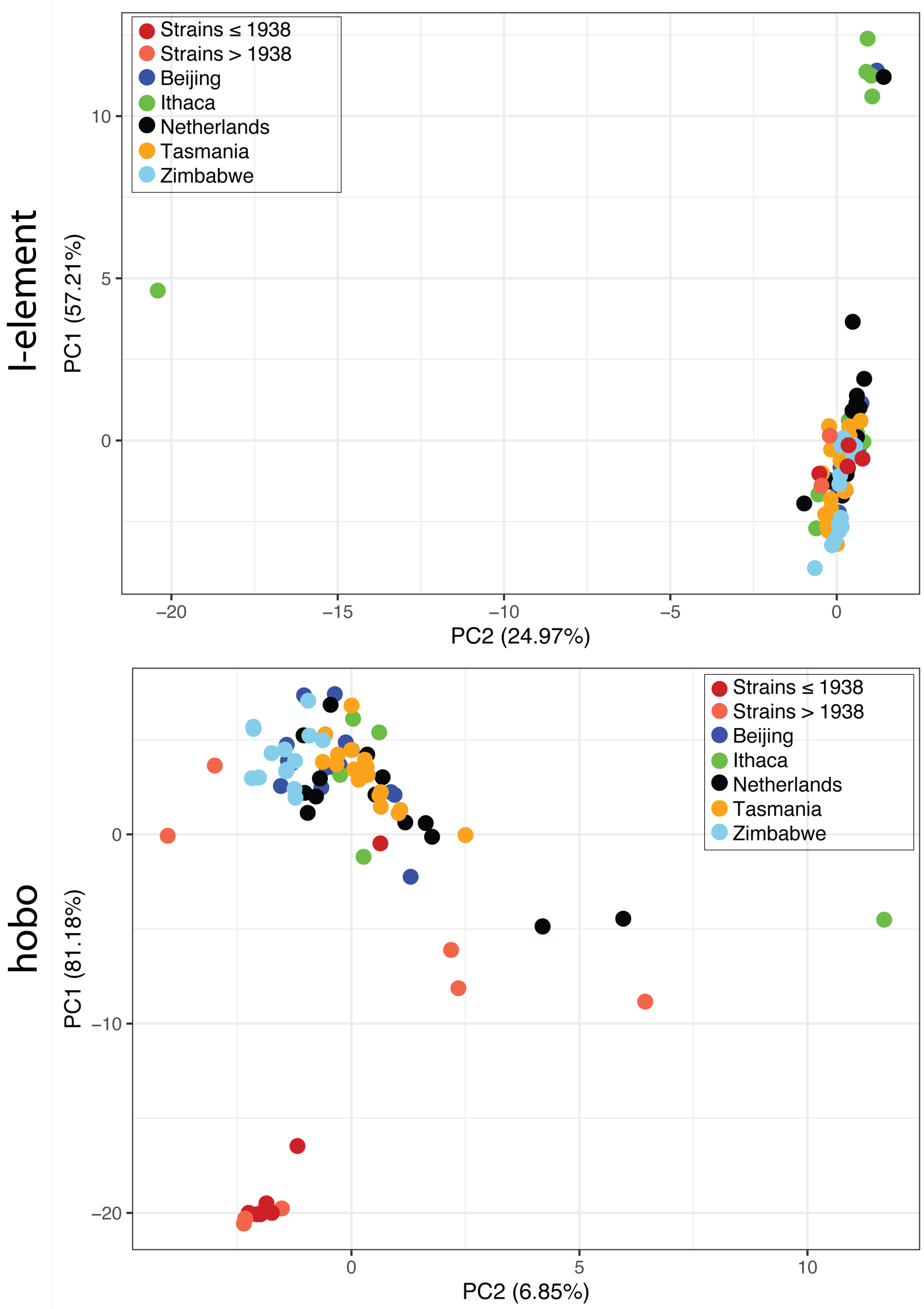

Figure 14: PCA based on the allele frequencies of SNPs in the I-element and hobo. Tasmanian populations cluster with strains from other geographic regions for both TEs.

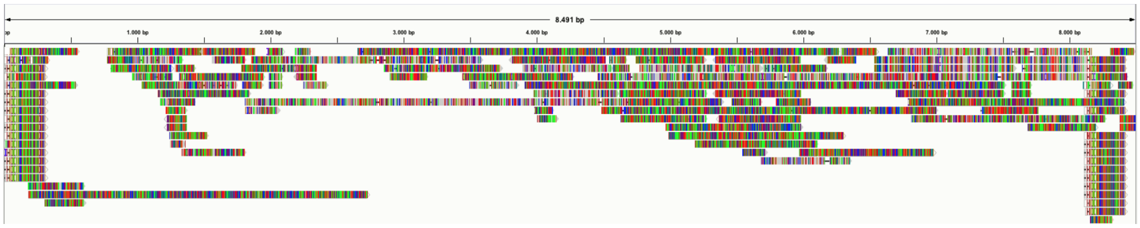

Figure 15: Alignment of all Tirant sequences annotated in Canton-S with the canonical Tirant sequence using permissive parameters. Colored lines represent SNPs with respect to the canonical Tirant. Note that a high sequence divergence can be found for all fragments over the entire sequence.

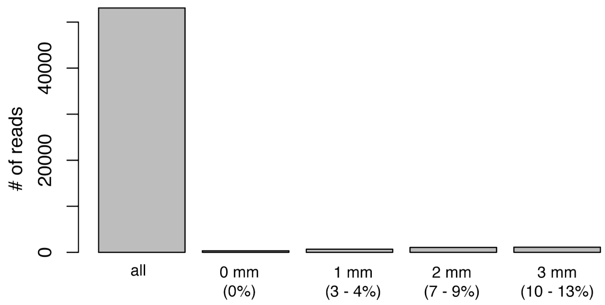

Figure 16: Number of piRNAs from heterochromatic Tirant insertions (all) mapping to the canonical Tirant sequence using different numbers of mismatches tolerated. The maximum divergence of the mapped piRNAs is shown in a brackets (based on the given number of mismatches and a piRNA length of 23-29nt).

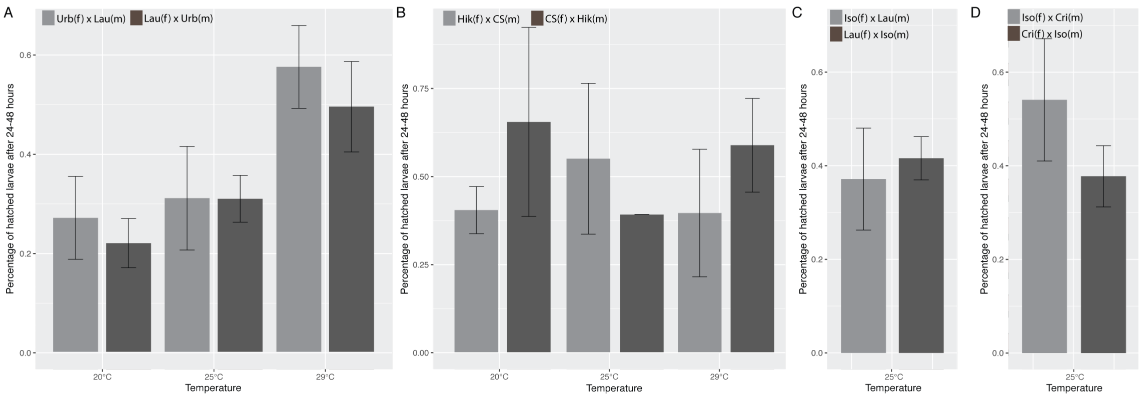

Figure 17: Fraction of hatched F2 eggs for reciprocal crosses between a strains having canonical Tirant insertions (Urb: Urbana-S, Hik: Hikone-R, Iso:Iso-1) and a strains not having canonical Tirant insertions (Lau: Lausanne-S, CS: Canton-S, Cri: Crimea). Crosses were performed at up to three different temperatures and three replicates were used for each cross. We did not detect significant differences in the abundance of hatched F2 eggs between the reciprocal crosses; Wilcoxon rank sum test; Urbana-S and Lausanne-S: $p_{20} = 0.4$, $p_{25} = 0.7$, $p_{29} = 0.4$; Hikone-R and Canton-S: $p_{20} = 0.4$, $p_{25} = 0.3$, $p_{29} = 0.4$; Iso-1 x Lausanne-S: $p_{25} = 1$; Iso-1 x Crimea: $p_{25} = 0.2$; m males, f females

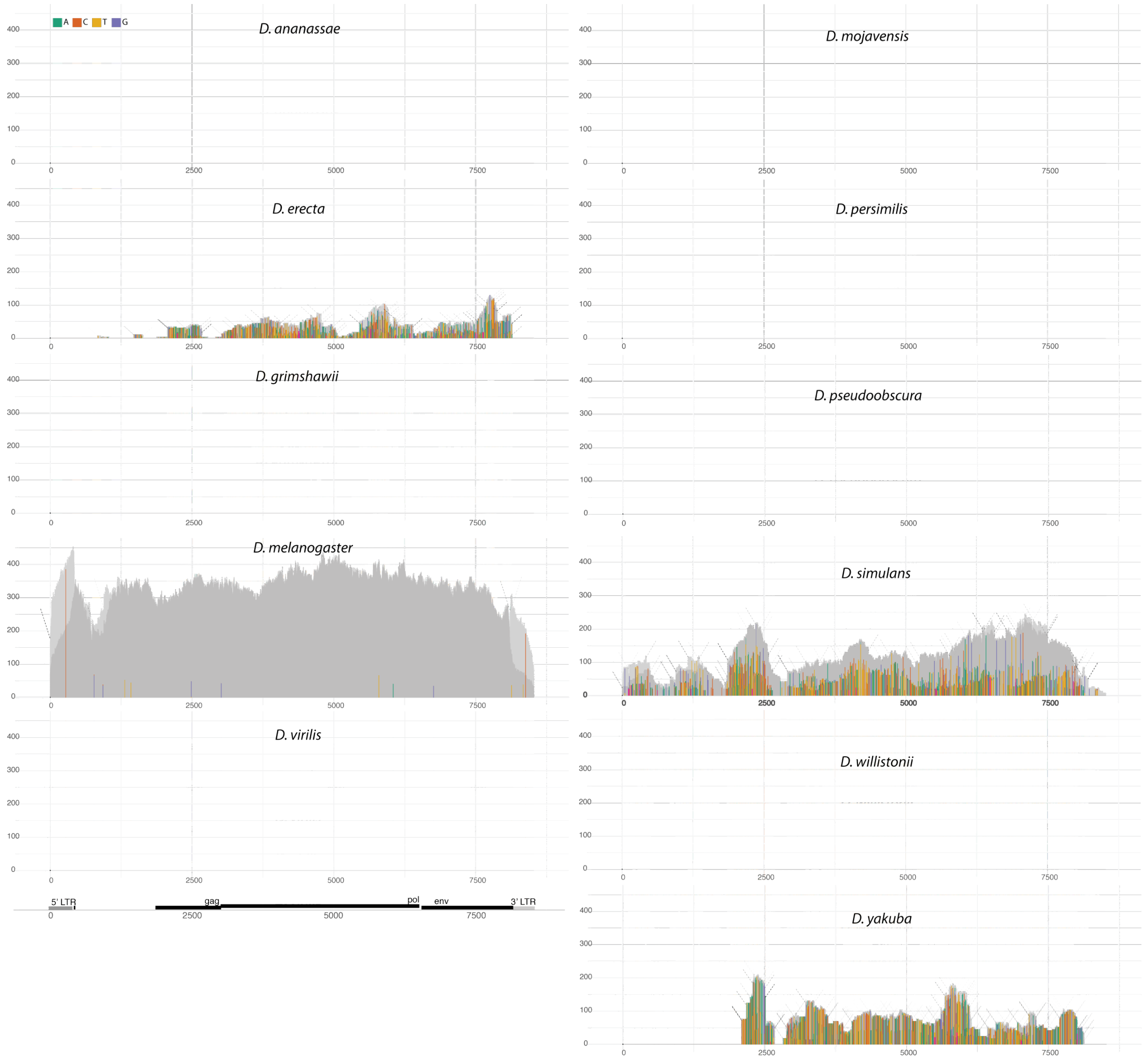

Figure 18: Abundance and diversity of Tirant sequences in 11 Drosophila species (*Drosophila* 12 Genomes Consortium, 2007). Tirant sequences can solely be found in the *Drosophila melanogaster* species subgroup. Note that solely *D. melanogaster* and *D. simulans* may have full-length insertions of Tirant. Furthermore, some insertions in *D. simulans* have a high similarity to the consensus sequence of Tirant (few SNPs in the upper regions of the DeviaTE plot).

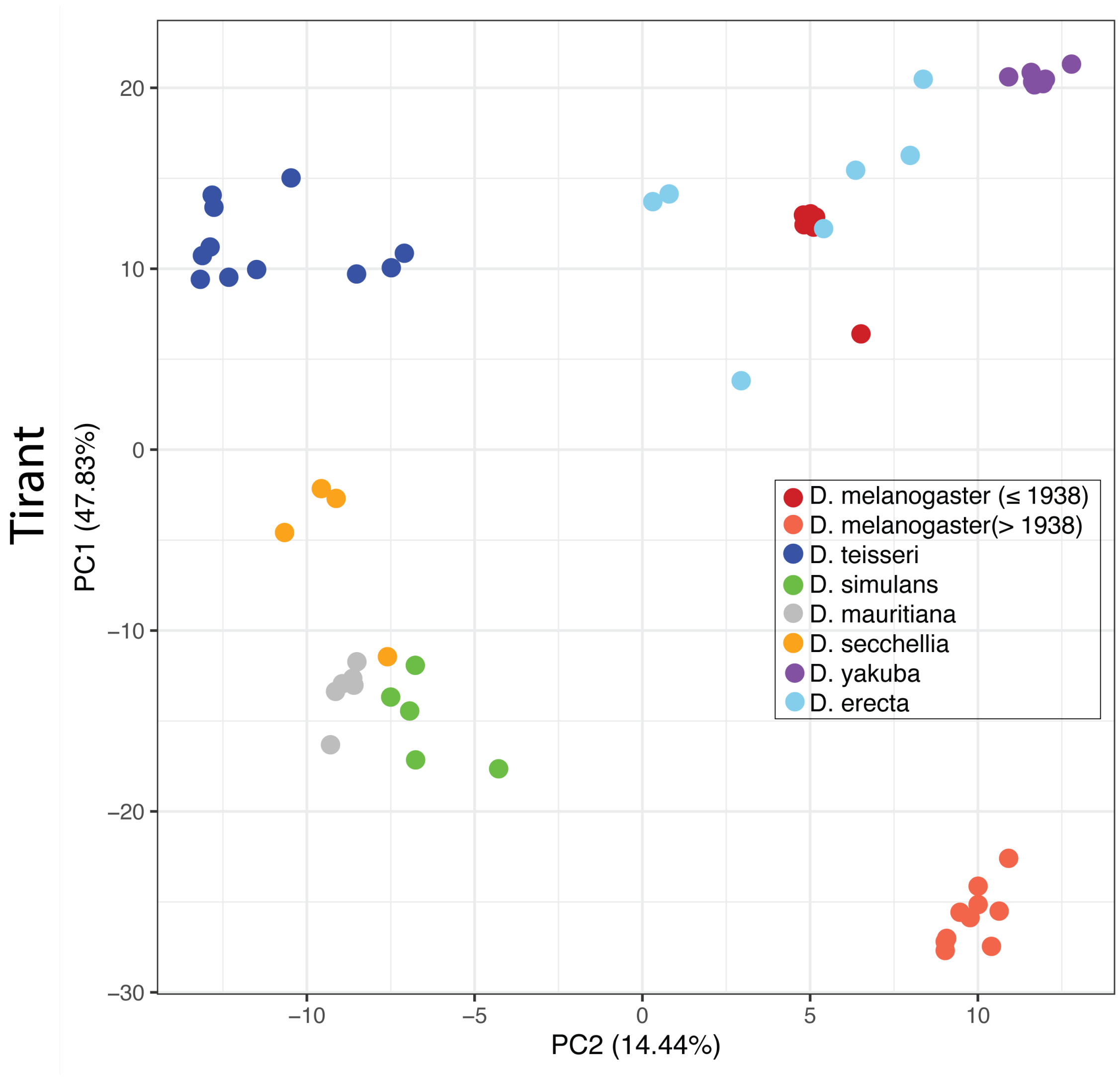

Figure 19: PCA based on the allele frequencies of SNPs in Tirant. Data are shown for several lines of different species from the *Drosophila melanogaster* species subgroup. Note that old lab strains of *D. melanogaster* cluster with *D. erecta* while more recently collected strain are closest to *D. simulans*.

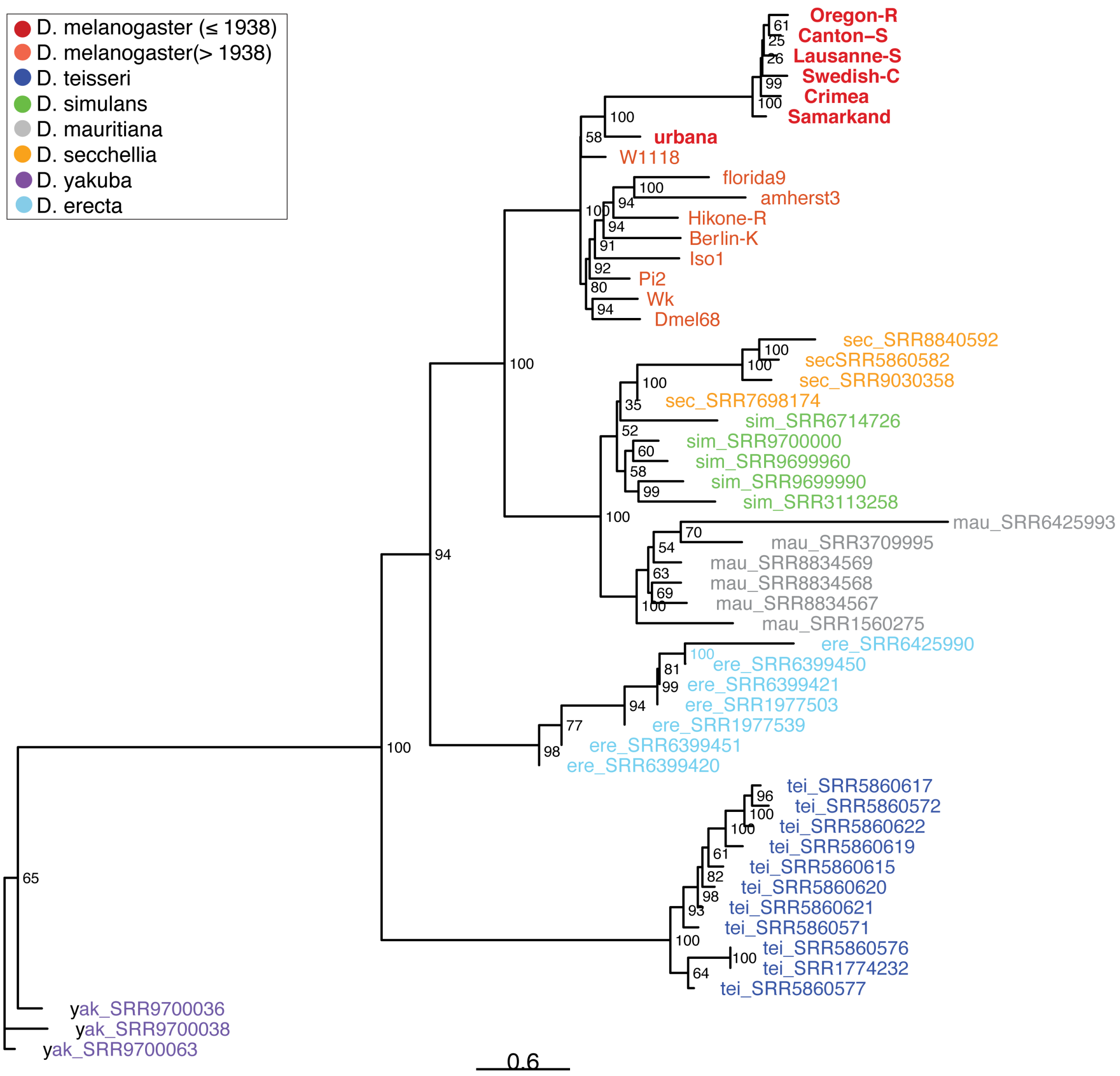

Figure 20: Phylogenetic tree calculated with PoMo based on the allele frequencies of SNPs in Tirant. Data are shown for several lines of different species from the *D. melanogaster* species subgroup. Note that the tree mostly follows the current species phylogeny of the *D. melanogaster* species subgroup (Obbard et al., 2012). *D. melanogaster* and *D. simulans* carry two distinct variants of Tirant sequences that are combined in this analysis. However, diverged Tirant sequences are most abundant in both species (82.55% of annotated Tirant sequences in *D. melanogaster* and 98.3% in *D. simulans*).

Supplementary tables

Table 1: Overview of the abundance of Tirant, I-element, hobo, and P-element sequences in different *D. melanogaster* strains. Strains are ordered by their estimated collection date. For each family and strain, we classified the TE content into three distinct categories: 'red' absence of any TE sequence, 'yellow' solely degraded TE sequences are present, 'green' non-degraded sequences, with a high similarity to the consensus sequence are present. Numbers in brackets represent the average coverage normalized to single-copy genes (≈ TE copy numbers per haploid genome). Strains sequenced in this work are marked by a star (*). † latest possible collection date was inferred from death of C. Bridges (1938), who collected the strain (Lindsley and Grell, 1968). coll. date collection date, FlyBase `https://flybase.org/`, NDSSC `https://www.drosophilaspecies.com/`

strain	coll. date	Tirant	I-ele.	hobo	P-ele.	location	source
Oregon-R	1925	~ (0.6)	~ (19.9)	~ (5.8)	− (0)	Oregon, USA	Lindsley and Grell 1968
Canton-S	1935	~ (0.9)	~ (19.5)	~ (5.9)	− (0)	Ohio, USA	Anxolabéhère *et al.* 1988
Samarkand	1936	~ (0.7)	~ (17.6)	~ (4.3)	− (0)	Samarkand, Uzbekistan	Lindsley and Grell 1968
Crimea*	1936	~ (0.5)	~ (18.5)	~ (4.5)	− (0)	Crimea, Eastern Europe	Anxolabéhère *et al.* 1988
Lausanne-S*	1938	~ (0.5)	~ (18.8)	~ (3.7)	− (0)	Wisconsin, USA	Lindsley and Grell 1968
Swedish-C*	<1938(1923)	~ (0.6)	+ (37.5)	+ (27.7)	~ (0.3)	Stockholm, Sweden	Lindsley and Grell 1968
Urbana-S*	<1938	+ (2.4)	~ (21.9)	~ (5.5)	− (0)	Illinois, USA	Bridges†, (Lindsley and Grell, 1968)
Berlin-K*	<1950	+ (6.6)	+ (32.1)	~ (3.6)	− (0)	Berlin, Germany	Ruebenbauer *et al.* 2008
Hikone-R*	1950-59	+ (6.2)	~ (17.9)	~ (5.8)	− (0)	Japan	Galindo *et al.* 1995
Florida-9*	<1952	+ (7.6)	+ (31.4)	+ (26.9)	+ (77.3)	Florida, USA	Lindsley and Grell 1968
Dmel68*	1954	+ (14.1)	+ (40.6)	+ (16.2)	− (0)	Israel	NDSSC
B1(BER1)	1954	+ (15.8)	+ (30.0)	~ (2.6)	− (0)	Bermuda	FlyBase
A3(BS1)	1954	+ (13.7)	+ (23.3)	~ (2.4)	− (0)	Barcelona, Spain	FlyBase
B2(CA1)	1954	+ (8.2)	+ (33.7)	~ (1.3)	− (0)	Capetown, South Africa	FlyBase
B3(QI2)	1954	+ (10.2)	+ (30.3)	+ (11.1)	− (0)	Israel	FlyBase
A2(BOG1)	1962	+ (18.2)	+ (40.7)	+ (19.5)	− (0)	Bogota, Colombia	FlyBase
A4(KSA2)	1963	+ (3.0)	+ (29.4)	~ (1.3)	− (0)	Koriba Dam, Zimbabwe	FlyBase
B4(RVC3)	1963	+ (15.8)	+ (44.1)	+ (64.7)	− (0)	California, USA	FlyBase
A5(VAG1)	1965	+ (7.7)	+ (31.4)	+ (10.9)	− (0)	Athens, Greece	FlyBase
A6(wild5B)	1966	+ (15.2)	+ (31.3)	+ (84.2)	− (0)	Georgia, USA	FlyBase
Harwich	1967	+ (5.5)	+ (55.2)	+ (12.9)	+ (60.1)	Massachusets, USA	NDSSC
Pi2*	1975	+ (8.8)	+ (31.7)	+ (98.9)	+ (39.7)	N.A.	Engels 1979
w1118*	<1987	+ (5.2)	+ (40.5)	+ (35.5)	− (0)	N.A.	first used by Black *et al.* 1987
AB8 (Sam;ry506)	N.A.	~ (0.5)	~ (28.1)	~ (2.5)	− (0)	N.A.	N.A.
wk*	N.A.	+ (10.4)	~ (18.0)	~ (4.7)	− (0)	N.A.	N.A.
Amherst-3*	N.A.	+ (11.8)	+ (35.2)	~ (4.7)	− (0)	Massachusets, USA	N.A.
Iso1	N.A.	+ (20.9)	+ (32.0)	+ (28.6)	− (0)	N.A.	N.A.

Table 2: The abundance of Tirant, I-element, hobo, and P-element sequences in different *D. melanogaster* strains estimated with two different approaches: i) with DeviaTE based on the coverage of single copy genes (scg) and ii) as the normalized number of reads mapping to each TE (rpm; reads per million).For the sampling data of strains see supplementary table 1

	DeviaTE with scg.				rpm			
strain	Tirant	I-element	hobo	P-element	Tirant	I-element	hobo	P-element
Oregon-R	0.6	19.9	5.8	0.0	43.04	820.95	126.72	0.025
Canton-S	0.9	19.5	5.9	0.0	43.64	747.89	126.67	0.0
Samarkand	0.7	17.6	4.3	0.0	46.63	715.08	92.29	0.025
Crimea*	0.5	18.5	4.5	0.0	37.44	639.57	85.52	0.0
Lausanne-S*	0.5	18.8	3.7	0.0	46.13	729.53	79.53	0.054
Swedish-C*	0.6	37.5	27.7	0.3	47.70	1461.67	506.78	6.33
Urbana-S*	2.4	21.9	5.5	0.0	149.03	809.59	113.07	0.076
Berlin-K*	6.6	32.1	3.6	0.0	341.16	1114.81	71.35	0.0
Hikone-R*	6.2	17.9	5.8	0.0	332.03	689.45	122.49	0.0
Florida-9*	7.6	31.4	26.9	77.3	425.74	1168.60	428.50	857.25
Dmel68*	14.1	40.6	16.2	0.0	538.57	1355.19	329.42	0.049
B1(BER1)	15.8	30.0	2.6	0.0	701.70	907.19	32.63	0.0
A3(BS1)	13.7	23.3	2.4	0.0	609.78	1306.47	356.14	0.0
B2(CA1)	8.2	33.7	1.3	0.0	348.53	697.04	27.95	0.0
B3(QI2)	10.2	30.3	11.1	0.0	483.64	1072.19	15.93	0.0
A2(BOG1)	18.2	40.7	19.5	0.0	832.94	967.37	15.76	0.0
A4(KSA2)	3.0	29.4	1.3	0.0	129.29	1012.27	209.47	0.0
B4(RVC3)	15.8	44.1	64.7	0.0	698.92	1408.05	1216.57	0.0
A5(VAG1)	7.7	31.4	10.9	0.0	349.46	1084.80	211.92	0.0
A6(wild5B)	15.2	31.3	84.2	0.0	720.72	1016.0	1645.18	0.0
Harwich	5.5	55.2	12.9	60.1	275.29	1953.82	232.75	955.57
Pi2*	8.8	31.7	98.9	39.7	334.27	1206.16	1632.15	566.52
w1118*	5.2	40.5	35.5	0.0	186.42	1456.42	703.03	0.0
AB8	0.5	28.1	2.5	0.0	13.74	805.10	31.17	0.0
wk*	10.4	18.0	4.7	0.0	457.35	632.20	94.52	0.062
Amherst-3*	11.8	35.2	4.7	0.0	629.89	1296.54	96.80	0.0
Iso1	20.9	32.0	28.6	0.0	1054.42	1231.07	440.42	0.0

Table 3: Position in Tirant (pos.), reference allele (ref.) and frequency of the reference allele for SNPs with notable allele frequency differences between Iso-1 and natural populations (GDL, DrosEU and Dros-RTEC). For an overview of all SNPs in Iso-1 and some GDL lines see supplementary fig. 11.

pos	refbase	Iso-1 (SRR1663590)	GDL-Other (SRR1663540)	GDL-Other (SRR1663560)	GDL-Other (SRR1663600)	DrosEU (SRR5647729)	DrosEU (SRR5647776)	Dros-RTEC (SRR3590550)	Dros-RTEC (SRR3939104)
231	C	0.948	0.013	0	0	0.011	0	0	0
2486	A	0.792	0	0	0	0.046	0.036	0.038	0.057
2873	G	0.937	0	0	0	0.054	0.027	0.028	0.073
3015	A	0.891	0	0.167	0	0.032	0.023	0.033	0.05
5794	C	0.908	0	0	0	0	0	0	0
5926	A	0.931	0.134	0.152	0.227	0.131	0.14	0.042	0.103
6046	G	0.882	0	0	0	0	0.012	0.053	0.047
8338	C	0.931	0	0	0	0.004	0	0	0.003

Table 4: Position in Tirant (pos), reference allele (ref.) and frequency of the reference allele for SNPs with notable allele frequency differences between populations from Tasmania and other geographic locations (GDL). For an overview of all SNPs in Tasmanian and non-Tasmanian populations see supplementary fig. 12.

pos	ref.	GDL-Tasm. (SRR1663590)	GDL-Tasm. (SRR1663591)	GDL-Tasm. (SRR1663592)	GDL-Other (SRR1663540)	GDL-Other (SRR1663560)	GDL-Other (SRR1663600)
276	T	0.071	0.04	0.097	0.93	0.336	0.873
3922	G	0.075	0.111	0.084	0.68	0.824	0.791
5092	T	0.396	0.682	0.923	1	1	1
6758	A	0.932	1	0.993	0.734	0.366	0.439
8383	T	0.068	0.066	0.137	0.871	0.354	0.788

Table 5: Number of dysgenic and not-dysgenic ovaries in the F1 of reciprocal crosses between strains having canonical Tirant insertions (Urbana-S, Hikone-R or Iso-1) and strains not having canonical Tirant insertions (Lausanne-S, Canton-S or Crimea). Crosses were performed at up to two temperatures and three replicates were used for each cross. The direction of the cross had no significant influence on the fraction of dysgenic ovaries at both temperatures (Cochran–Mantel–Haenszel test; Urbana-S x Lausanne-S: $p_{25} = 0.736$, $p_{29} = 0.742$; Hikone-R x Canton-S:$p_{29} = 0.9611$; Iso-1 x Lausanne-S: $p_{25} = 0.867$; Iso-1 x Crimea:$p_{25} = 0.994$).

female	male	temp.	rep.	not-dysgenic	dysgenic
Urbana-S	Lausanne-S	25°C	1	17	0
Urbana-S	Lausanne-S	25°C	2	13	0
Urbana-S	Lausanne-S	25°C	3	13	0
Urbana-S	Lausanne-S	29°C	1	12	1
Urbana-S	Lausanne-S	29°C	2	18	0
Urbana-S	Lausanne-S	29°C	3	18	0
Lausanne-S	Urbana-S	25°C	1	13	0
Lausanne-S	Urbana-S	25°C	2	15	0
Lausanne-S	Urbana-S	25°C	3	15	0
Lausanne-S	Urbana-S	29°C	1	18	0
Lausanne-S	Urbana-S	29°C	2	11	0
Lausanne-S	Urbana-S	29°C	3	15	0
Hikone-R	Canton-S	29°C	1	18	0
Hikone-R	Canton-S	29°C	2	16	0
Hikone-R	Canton-S	29°C	3	16	0
Canton-S	Hikone-R	29°C	1	18	0
Canton-S	Hikone-R	29°C	2	14	0
Canton-S	Hikone-R	29°C	3	16	0
Iso1	Lausanne-S	25°C	1	16	0
Iso1	Lausanne-S	25°C	2	17	0
Iso1	Lausanne-S	25°C	3	20	0
Lausanne-S	Iso1	25°C	1	18	0
Lausanne-S	Iso1	25°C	2	19	0
Lausanne-S	Iso1	25°C	3	19	0
Iso1	Crimea	25°C	1	19	0
Iso1	Crimea	25°C	2	14	0
Iso1	Crimea	25°C	3	19	0
Crimea	Iso1	25°C	1	16	0
Crimea	Iso1	25°C	2	17	0
Crimea	Iso1	25°C	3	19	0

Table 6: Publicly available short read data used in this work

Mackay et al. (2012)	SRR018294, SRR018305, SRR018517, SRR018521, SRR018574, SRR018580, SRR018582, SRR018593, SRR018601, SRR834538, SRR834536, SRR834540, SRR834542, SRR834550, SRR834548, SRR048925, SRR834549, SRR834524, SRR834525, SRR834528, SRR834529, SRR834531, SRR834532, SRR834533, SRR834534, SRR834535, SRR834504, SRR834530, SRR834510, SRR834505, SRR834506, SRR834507, SRR834513, SRR834515, SRR834516, SRR834544, SRR834539, SRR834554, SRR834520, SRR835025, SRR835023, SRR835028, SRR835026, SRR835029, SRR051592, SRR051594, SRR835024, SRR835027, SRR835031, SRR835032, SRR835033, SRR835037, SRR835035, SRR835036, SRR835038, SRR835040, SRR835041, SRR835042, SRR835045, SRR835046, SRR051896, SRR835081, SRR835101, SRR051905, SRR835082, SRR835083, SRR835085, SRR835099, SRR835088, SRR835089, SRR835091, SRR835092, SRR835093, SRR835094, SRR835095, SRR835051, SRR835052, SRR835057, SRR835053, SRR835064, SRR835065, SRR835066, SRR835070, SRR835067, SRR835068, SRR835071, SRR835074, SRR835075, SRR835076, SRR835078, SRR835080, SRR835084, SRR835069, SRR835073, SRR834546, SRR835103, SRR060062, SRR835100, SRR060098, SRR835090, SRR835058, SRR834537, SRR834543, SRR835030, SRR835049, SRR060821, SRR835072, SRR834541, SRR835096, SRR835054, SRR834521, SRR834517, SRR834545, SRR835050, SRR933564, SRR835223, SRR835228, SRR835236, SRR835242, SRR835252, SRR835256, SRR933571, SRR933572, SRR933575, SRR933577, SRR835333, SRR933578, SRR933580, SRR933582, SRR933585, SRR933586, SRR933587, SRR933588, SRR933591, SRR933595, SRR835338, SRR933596, SRR933597, SRR933598, SRR933600, SRR835247, SRR933563, SRR932121, SRR835221, SRR933566, SRR933569, SRR933570, SRR933573, SRR835331, SRR835326, SRR933581, SRR933589, SRR933593, SRR933594, SRR835341, SRR835345, SRR933599, SRR835349, SRR835329, SRR933583, SRR933592, SRR933601, SRR933579, SRR835343, SRR835939,
Lack et al. (2015)	SRR189040, SRR1686796, SRR1688222, SRR189389, SRR306623, SRR306611, SRR203502, SRR306616, SRR306618
Bergland et al. (2014)	SRR1525685, SRR1525694, SRR1525695, SRR1525696, SRR1525697, SRR1525698, SRR1525699, SRR1525768, SRR1525769, SRR1525770, SRR1525771, SRR1525772, SRR1525773, SRR1525774, SRR2006283
Kapun et al. (2020)	SRR5647729, SRR5647730, SRR5647731, SRR5647732, SRR5647733, SRR5647734, SRR5647735, SRR5647736, SRR5647737, SRR5647738, SRR5647739, SRR5647740, SRR5647741, SRR5647742, SRR5647743, SRR5647744, SRR5647745, SRR5647746, SRR5647747, SRR5647748, SRR5647749, SRR5647750, SRR5647751, SRR5647752, SRR5647753, SRR5647754, SRR5647755, SRR5647756, SRR5647757, SRR5647758, SRR5647759, SRR5647760, SRR5647761, SRR5647762, SRR5647763, SRR5647764, SRR5647765, SRR5647766, SRR5647767, SRR5647768, SRR5647769, SRR5647770, SRR5647771, SRR5647772, SRR5647773, SRR5647774, SRR5647775, SRR5647776
Machado et al. (2019)	SRR3590550, SRR3590551, SRR3590554, SRR3590555, SRR3590556, SRR3590557, SRR3590558, SRR3590559, SRR3590560, SRR3590561, SRR3590562, SRR3590563, SRR3939042, SRR3939043, SRR3939044, SRR3939045, SRR3939046, SRR3939047, SRR3939048, SRR3939049, SRR3939050, SRR3939051, SRR3939052, SRR3939054, SRR3939056, SRR3939057, SRR3939058, SRR3939059, SRR3939076, SRR3939077, SRR3939078, SRR3939080, SRR3939081, SRR3939082, SRR3939083, SRR3939084, SRR3939085, SRR3939086, SRR3939087, SRR3939088, SRR3939089, SRR3939091, SRR3939092, SRR3939093, SRR3939094, SRR3939095, SRR3939096, SRR3939097, SRR3939098, SRR3939099, SRR3939100, SRR3939101, SRR3939102, SRR3939103, SRR3939104, SRR8061818, SRR8061819
Grenier et al. (2015)	SRR1663528, SRR1663529, SRR1663530, SRR1663531, SRR1663532, SRR1663533, SRR1663534, SRR1663535, SRR1663536, SRR1663537, SRR1663538, SRR1663539, SRR1663540, SRR1663541, SRR1663542, SRR1663543, SRR1663544, SRR1663545, SRR1663546, SRR1663547, SRR1663548, SRR1663549, SRR1663550, SRR1663551, SRR1663552, SRR1663553, SRR1663554, SRR1663555, SRR1663556, SRR1663557, SRR1663558, SRR1663559, SRR1663560, SRR1663561, SRR1663562, SRR1663563, SRR1663564, SRR1663565, SRR1663566, SRR1663567, SRR1663568, SRR1663569, SRR1663570, SRR1663571, SRR1663572, SRR1663573, SRR1663574, SRR1663575, SRR1663576, SRR1663577, SRR1663578, SRR1663579, SRR1663580, SRR1663581, SRR1663582, SRR1663583, SRR1663584, SRR1663585, SRR1663586, SRR1663587, SRR1663588, SRR1663589, SRR1663590, SRR1663591, SRR1663592, SRR1663593, SRR1663594, SRR1663595, SRR1663596, SRR1663597, SRR1663598, SRR1663599, SRR1663600, SRR1663601, SRR1663602, SRR1663603, SRR1663604, SRR1663605, SRR1663606, SRR1663607, SRR1663608, SRR1663609, SRR1663610, SRR1663611
Wierzbicki et al. (2020)	SRR11460805, SRR11460802, SRR11460799
Jakšić et al. (2017)	SRR5851905, SRR5851906
Garrigan et al. (2014)	SRR1560275
Turissini et al. (2015)	SRR1774232
Hill et al. (2016)	SRR3113258
Schrider et al. (2018)	SRR5860571, SRR5860572, SRR5860576, SRR5860577, SRR5860582
Lanno et al. (2019)	SRR5860615, SRR5860617, SRR5860619, SRR5860620, SRR5860621, SRR5860622
Miller et al. (2018)	SRR6425993
Kang et al. (2019)	SRR6714726
Melvin et al. (2018)	SRR7698174
Meany et al. (2019)	SRR8834567, SRR8834568, SRR8834569
Cooper et al. (2019)	SRR8840592
Garrigan et al. (2012)	SRR9030358, SRR9030360
Rogers et al. (2014)	SRR9699990, SRR9700000
Stewart and Rogers (2019)	SRR9700024, SRR9700028, SRR9700036, SRR9700038, SRR9700041, SRR9700049, SRR9700063

Table 7: Matches between the consensus sequence of Tirant and a long-read based assembly of *D. simulans* (strain w^{XD1}) (Chakraborty et al., 2020). Consecutive matches (having the same ID) from position 1(2) to 8,526 (i.e. the length of Tirant) of the query represent full-length insertions of Tirant. . For each hit we show the divergence in percent (div.) and the position in the reference genome and in the query sequence (chr, chromosome). The average divergence of the three reported insertions from the consensus sequence of Tirant is $d_1 = 1.97\%$, $d_2 = 1.56\%$, $d_3 = 1.60$;

			reference genome		query (Tirant)	
ID	div.	chr.	start	end	start	end
1	1.4	2R	3,374,847	3,376,593	8,526	6,797
1	1.3	2R	3,376,594	3,376,982	6,735	6,347
1	1.8	2R	3,376,967	3,379,835	3,818	948
1	3.8	2R	3,379,538	3,380,475	934	2
2	1.3	X	21,362,067	21,363,796	8,526	6,797
2	1.3	X	21,363,793	21,365,739	6,739	4,793
2	1.5	X	21,365,737	21,369,723	4,760	744
2	2.6	X	21,369,245	21,370,341	1,116	1
3	1.6	Y_7	26,081	27,810	8,526	6,797
3	1.3	Y_7	27,811	33,493	6,735	1,050
3	3.7	Y_7	33,298	34,131	832	2

References

Bergland, A. O., Behrman, E. L., O'Brien, K. R., Schmidt, P. S., and Petrov, D. A. (2014). Genomic Evidence of Rapid and Stable Adaptive Oscillations over Seasonal Time Scales in *Drosophila*. *PLoS Genetics*, 10(11).

Chakraborty, M., Chang, C.-H., Khost, D., Vedanayagam, J., Adrion, J. R., Liao, Y., Montooth, K. L., Meiklejohn, C. D., Larracuente, A. M., and Emerson, J. J. (2020). Evolution of genome structure in the *Drosophila simulans* species complex. *bioRxiv*.

Chakraborty, M., Emerson, J. J., Macdonald, S. J., and Long, A. D. (2019). Structural variants exhibit widespread allelic heterogeneity and shape variation in complex traits. *Nature Communications*, 10(1):419275.

Cooper, J. C., Guo, P., Bladen, J., and Phadnis, N. (2019). A triple-hybrid cross reveals a new hybrid incompatibility locus between *D. melanogaster* and *D. sechellia*. *bioRxiv*, page 590588.

Drosophila 12 Genomes Consortium (2007). Evolution of genes and genomes on the *Drosophila* phylogeny. *Nature*, 450(7167):203–18.

Garrigan, D., Kingan, S. B., Geneva, A. J., Andolfatto, P., Clark, A. G., Thornton, K. R., and Presgraves, D. C. (2012). Genome sequencing reveals complex speciation in the *Drosophila simulans* clade. *Genome Research*, 22(8):1499–1511.

Garrigan, D., Kingan, S. B., Geneva, A. J., Vedanayagam, J. P., and Presgraves, D. C. (2014). Genome diversity and divergence in *Drosophila mauritiana*: Multiple signatures of faster X evolution. *Genome Biology and Evolution*, 6(9):2444–2458.

Grenier, J. K., Roman Arguello, J., Moreira, M. C., Gottipati, S., Mohammed, J., Hackett, S. R., Boughton, R., Greenberg, A. J., and Clark, A. G. (2015). Global diversity lines-a five-continent reference panel of sequenced *Drosophila melanogaster* strains. *G3: Genes, Genomes, Genetics*, 5(4):593–603.

Hill, T., Schlötterer, C., and Betancourt, A. J. (2016). Hybrid Dysgenesis in *Drosophila simulans* Associated with a Rapid Invasion of the P-Element. *PLoS Genetics*, 12(3):1–17.

Hoskins, R. A., Carlson, J. W., Wan, K. H., Park, S., Mendez, I., Galle, S. E., Booth, B. W., Pfeiffer, B. D., George, R. A., Svirskas, R., et al. (2015). The Release 6 reference sequence of the *Drosophila melanogaster* genome. *Genome research*, 25(3):445–458.

Jakšić, A. M., Kofler, R., and Schlötterer, C. (2017). Regulation of transposable elements: Interplay between TE-encoded regulatory sequences and host-specific trans-acting factors in *Drosophila melanogaster*. *Molecular Ecology*, 26(19):5149–5159.

Kang, L., Rashkovetsky, E., Michalak, K., Garner, H. R., Mahaney, J. E., Rzigalinski, B. A., Korol, A., Nevo, E., and Michalak, P. (2019). Genomic divergence and adaptive convergence in *Drosophila simulans* from Evolution Canyon, Israel. *Proceedings of the National Academy of Sciences*, 116(24):11839 – 11844.

Kapun, M., Barrón, M. G., Staubach, F., Obbard, D. J., Wiberg, R. A. W., Vieira, J., Goubert, C., Rota-Stabelli, O., Kankare, M., Bogaerts-Márquez, M., et al. (2020). Genomic Analysis of European *Drosophila melanogaster* Populations Reveals Longitudinal Structure, Continent-Wide Selection, and Previously Unknown DNA Viruses. *Molecular Biology and Evolution*, 37(9):2661–2678.

Lack, J. B., Cardeno, C. M., Crepeau, M. W., Taylor, W., Corbett-Detig, R. B., Stevens, K. A., Langley, C. H., and Pool, J. E. (2015). The *Drosophila* genome nexus: a population genomic resource of 623 *Drosophila melanogaster* genomes, including 197 from a single ancestral range population. *Genetics*, 199(4):1229–1241.

Lanno, S. M., Shimshak, S. J., Peyser, R. D., Linde, S. C., and Coolon, J. D. (2019). Investigating the role of Osiris genes in *Drosophila sechellia* larval resistance to a host plant toxin. *Ecology and Evolution*, 9(4):1922–1933.

Lindsley, D. H. and Grell, E. H. (1968). *Genetic variations of Drosophila melanogaster.* Carnegie Institute of Washington Publication.

Machado, H. E., Bergland, A. O., Taylor, R., Tilk, S., Behrman, E., Dyer, K., Fabian, D. K., Flatt, T., González, J., Karasov, T. L., et al. (2019). Broad geographic sampling reveals predictable, pervasive, and strong seasonal adaptation in *Drosophila*. *bioRxiv*, page 337543.

Mackay, T. F., Richards, S., Stone, E. A., Barbadilla, A., Ayroles, J. F., Zhu, D., Casillas, S., Han, Y., Magwire, M. M., Cridland, J. M., et al. (2012). The *Drosophila melanogaster* genetic reference panel. *Nature*, 482(7384):173–178.

Meany, M. K., Conner, W. R., Richter, S. V., Bailey, J. A., Turelli, M., and Cooper, B. S. (2019). Loss of cytoplasmic incompatibility and minimal fecundity effects explain relatively low Wolbachia frequencies in *Drosophila mauritiana*. *Evolution*, 73(6):1278–1295.

Melvin, R. G., Lamichane, N., Havula, E., Kokki, K., Soeder, C., Jones, C. D., and Hietakangas, V. (2018). Natural variation in sugar tolerance associates with changes in signaling and mitochondrial ribosome biogenesis. *eLife*, 7:e40841.

Miller, D. E., Staber, C., Zeitlinger, J., and Hawley, R. S. (2018). Highly Contiguous Genome Assemblies of 15 *Drosophila* Species Generated Using Nanopore Sequencing . *G3; Genes|Genomes|Genetics*, 8(10):3131–3141.

Obbard, D. J., Maclennan, J., Kim, K.-W., Rambaut, A., O'Grady, P. M., and Jiggins, F. M. (2012). Estimating Divergence Dates and Substitution Rates in the *Drosophila* Phylogeny. *Molecular Biology and Evolution*, 29(11):3459–3473.

Riddle, N. C., Minoda, A., Kharchenko, P. V., Alekseyenko, A. A., Schwartz, Y. B., Tolstorukov, M. Y., Gorchakov, A. A., Jaffe, J. D., Kennedy, C., Linder-Basso, D., et al. (2011). Plasticity in patterns of histone modifications and chromosomal proteins in *Drosophila* heterochromatin. *Genome Research*, 21(2):147–163.

Rogers, R. L., Cridland, J. M., Shao, L., Hu, T. T., Andolfatto, P., and Thornton, K. R. (2014). Landscape of standing variation for tandem duplications in *Drosophila yakuba* and *Drosophila simulans*. *Molecular biology and evolution*, 31(7):1750–1766.

Schrider, D., Ayroles, J., Matute, D., and Kern, A. (2018). Supervised machine learning reveals introgressed loci in the genomes of *Drosophila simulans* and *D. sechellia*. *PLOS Genetics*, 14(4):170670.

Stewart, N. B. and Rogers, R. L. (2019). Chromosomal rearrangements as a source of new gene formation in *Drosophila yakuba*. *PLOS Genetics*, 15(9):e1008314.

Turissini, D. A., Liu, G., David, J. R., and Matute, D. R. (2015). The evolution of reproductive isolation in the *Drosophila yakuba* complex of species. *Journal of Evolutionary Biology*, 28(3):557–575.

Wierzbicki, F., Schwarz, F., Cannalonga, O., and Kofler, R. (2020). Generating high quality assemblies for genomic analysis of transposable elements. *bioRxiv*.
