# Supplementary figures and images for: Tirant Stealthily Invaded Natural Drosophila melanogaster Populations during the Last Century
Source: Mol Biol Evol. 2020 Nov 28;38(4):1482–97. doi: 10.1093/molbev/msaa308 (PMC8042734; doi:10.1093/molbev/msaa308)

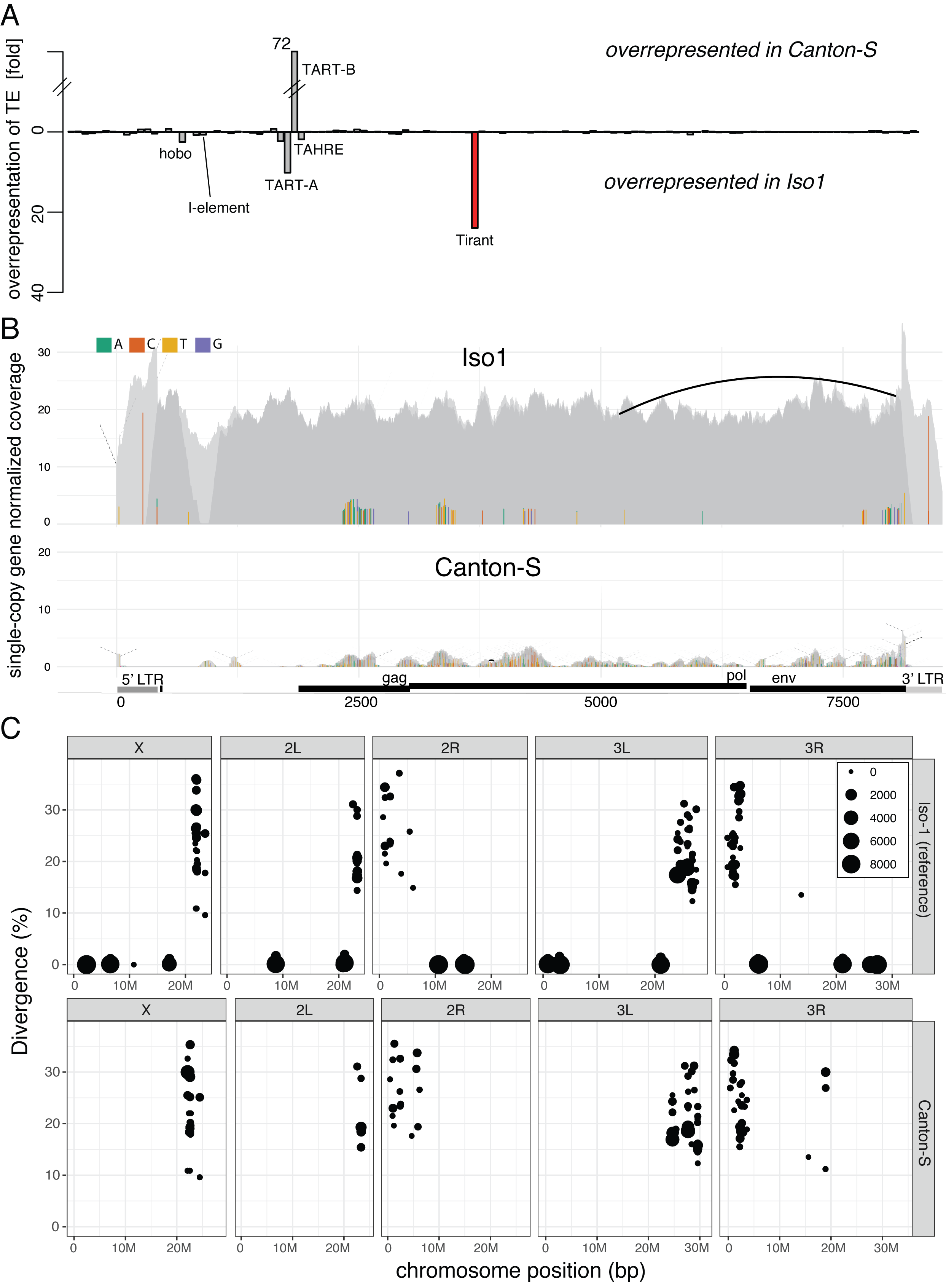

Supplement: msaa308_Supplementary_Data [file msaa308_supplementary_data.zip › Fig1_COMB_TElandscape_genome_piRNAs_Iso1_CantonS_v1_MODIFIED_deviaTE_Path0_05mm_nopiRNAs.png]

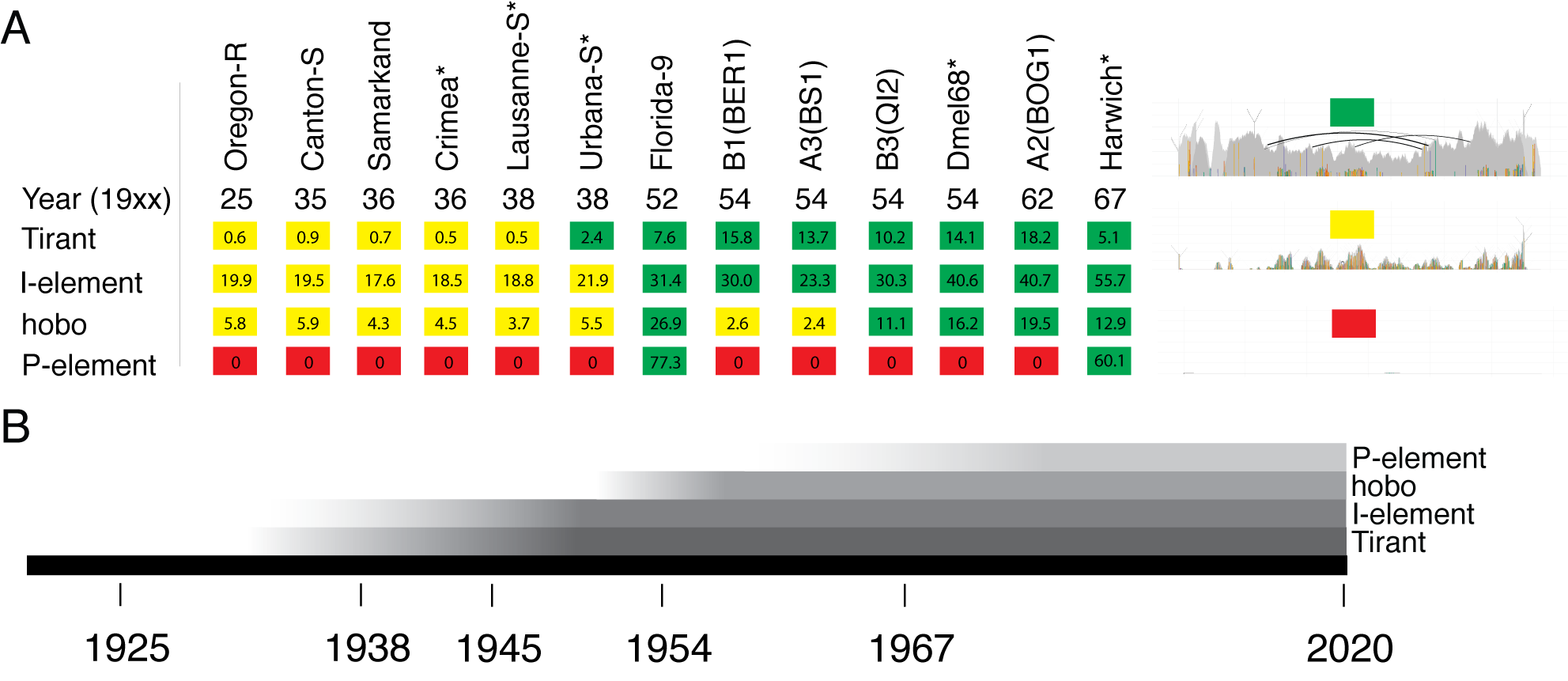

Supplement: msaa308_Supplementary_Data [file msaa308_supplementary_data.zip › Fig2.png]

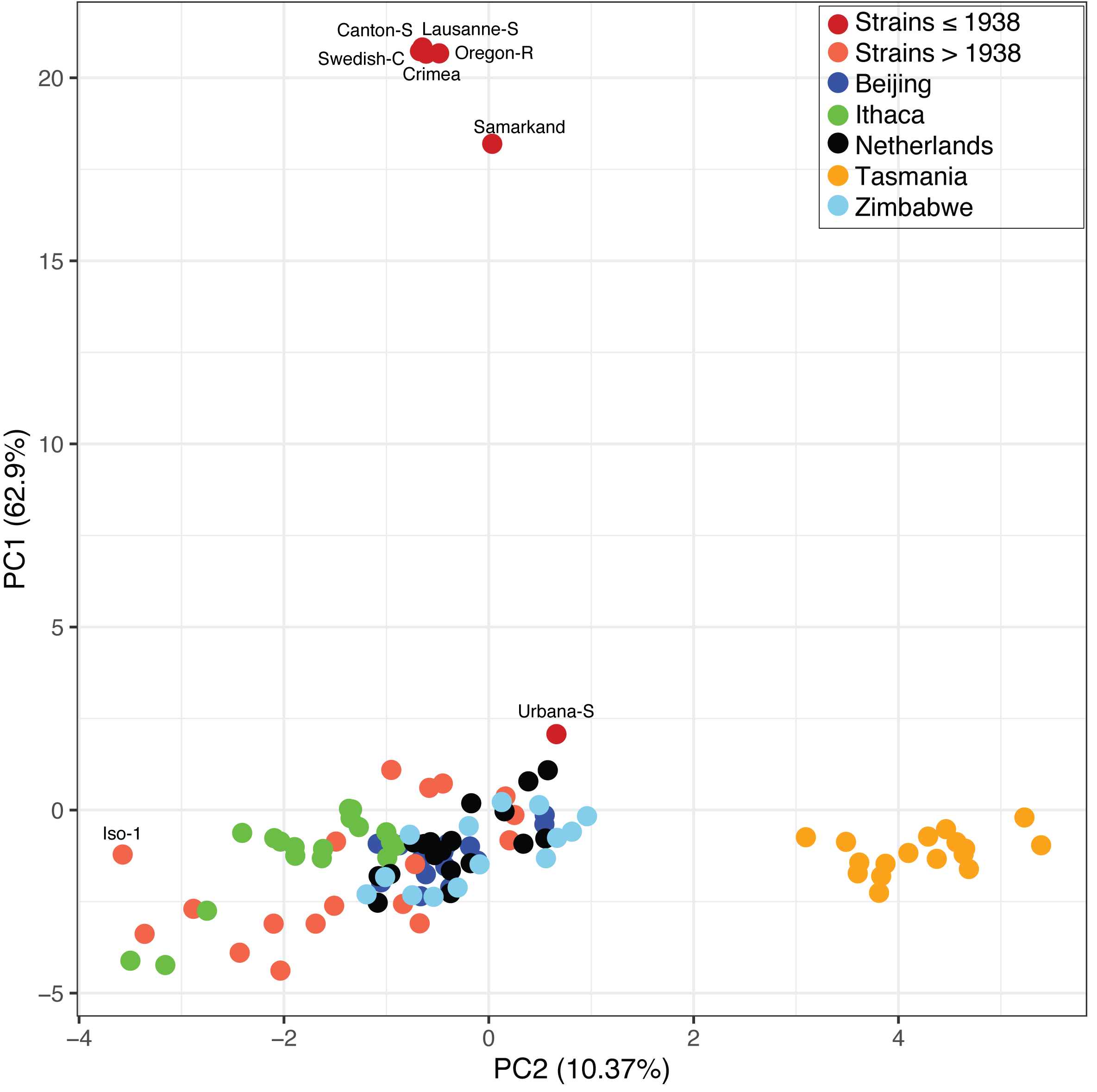

Supplement: msaa308_Supplementary_Data [file msaa308_supplementary_data.zip › Fig3_PCA_GDL_and_labstrains_colors_somelabels_transformed_v2.png]

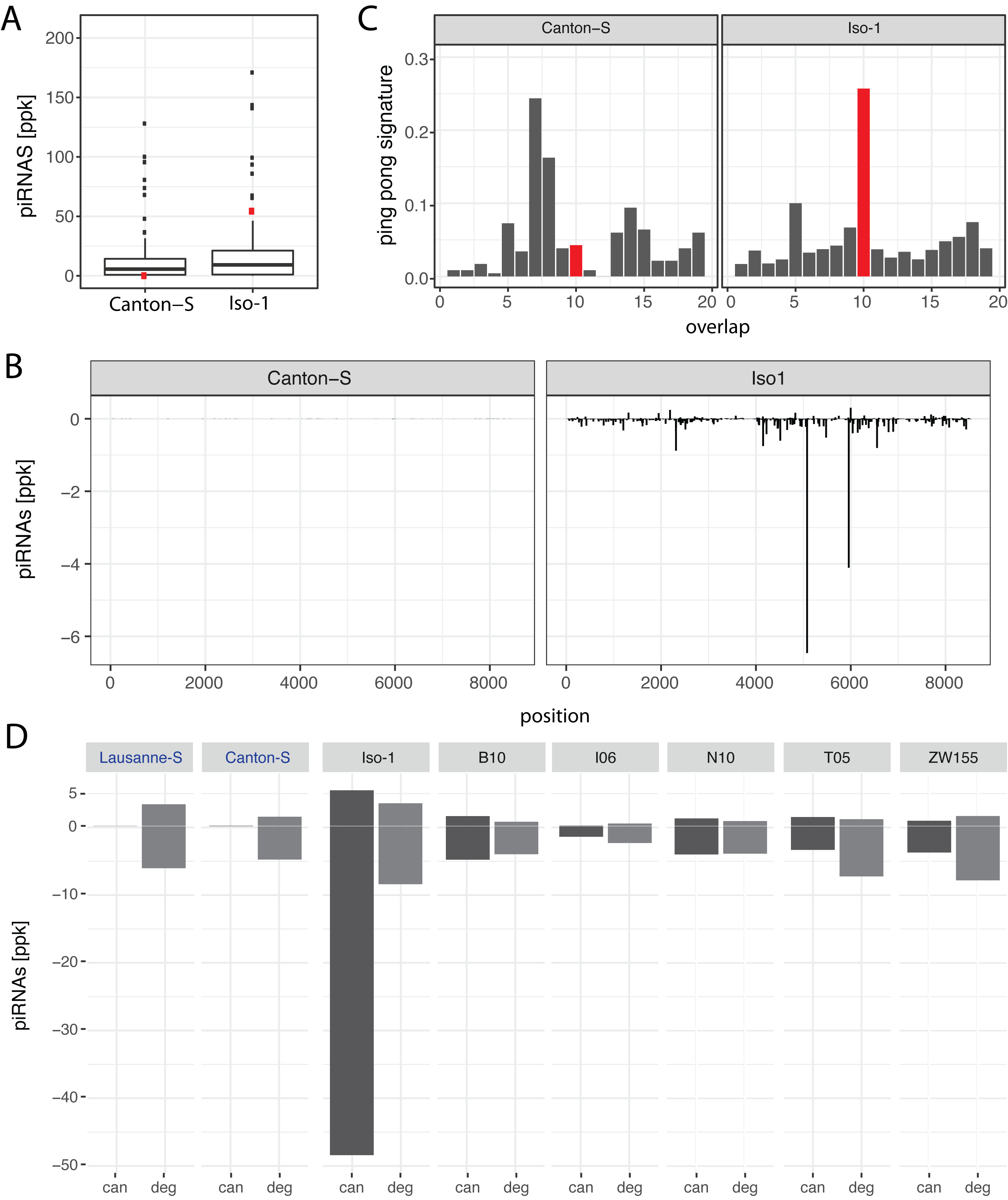

Supplement: msaa308_Supplementary_Data [file msaa308_supplementary_data.zip › Fig4_piRNAs.png]
